# Single-atomic-site platinum steers photogenerated charge carrier lifetime of hematite nanoflakes for photoelectrochemical water splitting

**DOI:** 10.1038/s41467-023-38343-6

**Published:** 2023-05-08

**Authors:** Rui-Ting Gao, Jiangwei Zhang, Tomohiko Nakajima, Jinlu He, Xianhu Liu, Xueyuan Zhang, Lei Wang, Limin Wu

**Affiliations:** 1 College of Chemistry and Chemical Engineering, College of Energy Material and Chemistry, Inner Mongolia University, Hohhot, 010021 China; 2 Advanced Manufacturing Research Institute, National Institute of Advanced Industrial Science and Technology, Tsukuba Central 5, 1-1-1 Higashi, Tsukuba, Ibaraki 305-8565 Japan; 3 College of Chemistry and Chemical Engineering, Inner Mongolia University, Hohhot, 010021 China; 4 Key Laboratory of Materials Processing and Mold, Ministry of Education, Zhengzhou University, Zhengzhou, 450002 China; 5 State Key Laboratory of Chemo/Biosensing and Chemometrics, College of Chemistry and Chemical Engineering, Hunan University, Changsha, 410082 China; 6 Department of Materials Science and State Key Laboratory of Molecular Engineering of Polymers, Fudan University, Shanghai, 200433 China

**Keywords:** Photocatalysis, Photocatalysis, Electron transfer

## Abstract

Although much effort has been devoted to improving photoelectrochemical water splitting of hematite (α-Fe_2_O_3_) due to its high theoretical solar-to-hydrogen conversion efficiency of 15.5%, the low applied bias photon-to-current efficiency remains a huge challenge for practical applications. Herein, we introduce single platinum atom sites coordination with oxygen atom (Pt-O/Pt-O-Fe) sites into single crystalline α-Fe_2_O_3_ nanoflakes photoanodes (SAs Pt:Fe_2_O_3_-Ov). The single-atom Pt doping of α-Fe_2_O_3_ can induce few electron trapping sites, enhance carrier separation capability, and boost charge transfer lifetime in the bulk structure as well as improve charge carrier injection efficiency at the semiconductor/electrolyte interface. Further introduction of surface oxygen vacancies can suppress charge carrier recombination and promote surface reaction kinetics, especially at low potential. Accordingly, the optimum SAs Pt:Fe_2_O_3_-Ov photoanode exhibits the photoelectrochemical performance of 3.65 and 5.30 mA cm^−2^ at 1.23 and 1.5 V_RHE_, respectively, with an applied bias photon-to-current efficiency of 0.68% for the hematite-based photoanodes. This study opens an avenue for designing highly efficient atomic-level engineering on single crystalline semiconductors for feasible photoelectrochemical applications.

## Abstract (web summary)

The achievable photocurrent of hematite, α-Fe_2_O_3_, is typically limited far below its theoretical limit. Here, the authors engineer single Pt atomic sites with surface oxygen vacancies into hematite photoanodes, which leads to enhanced photoelectrochemical water splitting.

## Introduction

Photoelectrochemical (PEC) water splitting has attracted great promises in recent years for sustainable hydrogen production^1–5^, in which the fabrication of the suitable semiconductor photoanodes with sufficient light absorption and efficient charge carrier transport is becoming increasingly important for achieving high solar-to-hydrogen (STH) conversion efficiency^6–10^. Many metal oxide semiconductors such as TiO_2_^11–13^, α-Fe_2_O_3_^14–16^, BiVO_4_^17–19^, and WO_3_^20–22^, have been considered as promising candidates owing to their availability, facile preparation, and oxidative stability. Especially, hematite (α-Fe_2_O_3_), a n-type semiconductor with a small band gap of ~2.1 eV, can absorb a large portion of the solar spectrum and allow a theoretical STH efficiency of 15.5% under standard sunlight illumination^23–26^. However, its short hole diffusion length, poor charge carrier conductivity, and sluggish oxygen evolution reaction kinetics limit the photocurrent of α-Fe_2_O_3_ far below its theoretical value of 12.4 mA cm^−2^^27,28^. Especially, the low electron mobility (~10^−2^ cm^2^ V^−1^ s^−1^) as one of the main intrinsic drawbacks significantly impedes its PEC performance^29–31^.

To enhance the electron mobility of photoelectrode, doping elements (e.g., Ti^32–34^, Sn^35–37^, Zr^38^, La^39^, Ta^27,40^, B^41^, and P^31^) have been adapted to substantially ameliorate the photo-efficiency of α-Fe_2_O_3_. For example, nonmetallic P doping has a superior activity owing to the strong covalent interaction between P and O, which boosted fast electron carriers and avoided the formation of deep electron trapping sites in α-Fe_2_O_3_^31^. Although these doping elements can improve more or less the electrical conductivity and charge transfer of α-Fe_2_O_3_, all the dopants in the photoelectrodes reported so far are clusters or bigger than clusters. As a result, not only the improvement of PEC is limited, but also the band bending caused by these clusters decreases the width of space-charge layer which limits the number of the carriers inside of the layer. In addition, these traditional dopants have not much influence on onset potential (generally located at 0.8–1.0 V_RHE_). The onset potential further influences the applied bias photon-to-current efficiency (ABPE) of α-Fe_2_O_3_ due to the surface and bulk trap states^3,42^, the high onset potential causing to the low ABPE. Therefore, additional strategies are desirable to improve the charge transfer efficiency and ABPE value.

Recently, engineering single atom catalysts have been widely used to enhance the oxygen evolution reaction performances. Unsaturated coordination environments of single atoms often function as active sites, influencing the catalytic performances^43,44^. However, most single atoms are incorporated into amorphous catalyst layers, and no reports are related to single atom doping semiconductors. For example, atomically dispersed Ni-N_4_ sites coordinated with oxygen atom have promoted photogenerated charge separation and thus improved PEC performance of BiVO_4_^45^. The single atom catalysts grown on an amorphous support act as the charge transfer layer, and the construction of single-atomic Ni-N_4_-O moiety is located at the interface of photoelectrode/oxygen evolution cocatalyst (OEC). In fact, more serious recombination usually happens in the bulk materials. To this end, it is highly desirable to design single metal atoms doped into photoelectrode to efficiently transfer carrier from the bulks to the photoelectrode/electrolyte interfaces, and improve the OER and PEC performances.

Herein, we develop a versatile strategy to engineer single platinum atom sites coordinated with an oxygen atom (Pt-O) incorporated into single crystalline hematite photoanodes by using 2,2-bipyridine as the ligand to chelate Pt cations, followed by the inert atmosphere treatment to remove the ligand. The construction of single atomic Pt-O coordination in Fe_2_O_3_ nanoflakes (SAs Pt:Fe_2_O_3_) owns few deep electron trapping sites in hematite. Accordingly, the SAs Pt:Fe_2_O_3_ with the surface oxygen vacancies can achieve the photocurrent density of 3.65 mA cm^−2^ at 1.23 V_RHE_ with a ABPE of 0.68%, which is more than double most of the previously reported values of the doped Fe_2_O_3_-based photoanodes (Supplementary Table 1), and even superior to the cocatalysts decorated Fe_2_O_3_-based photoanodes. X-ray absorption fine structure analysis, time-resolved spectroscopic investigation, and time domain density functional theory and nonadiabatic molecular dynamics calculations have demonstrated that the improved PEC activity can be ascribed to the construction of the single-atomic Pt-O and Pt-O-Fe in Fe_2_O_3_, which facilitates the hole transfer from Fe_2_O_3_ bulk to interface, extends the charge carrier lifetime, and accelerates the reaction kinetics for PEC water splitting.

## Results

### Electronic structure of Fe_2_O_3_ and Pt doped Fe_2_O_3_

**Fig. 1:**
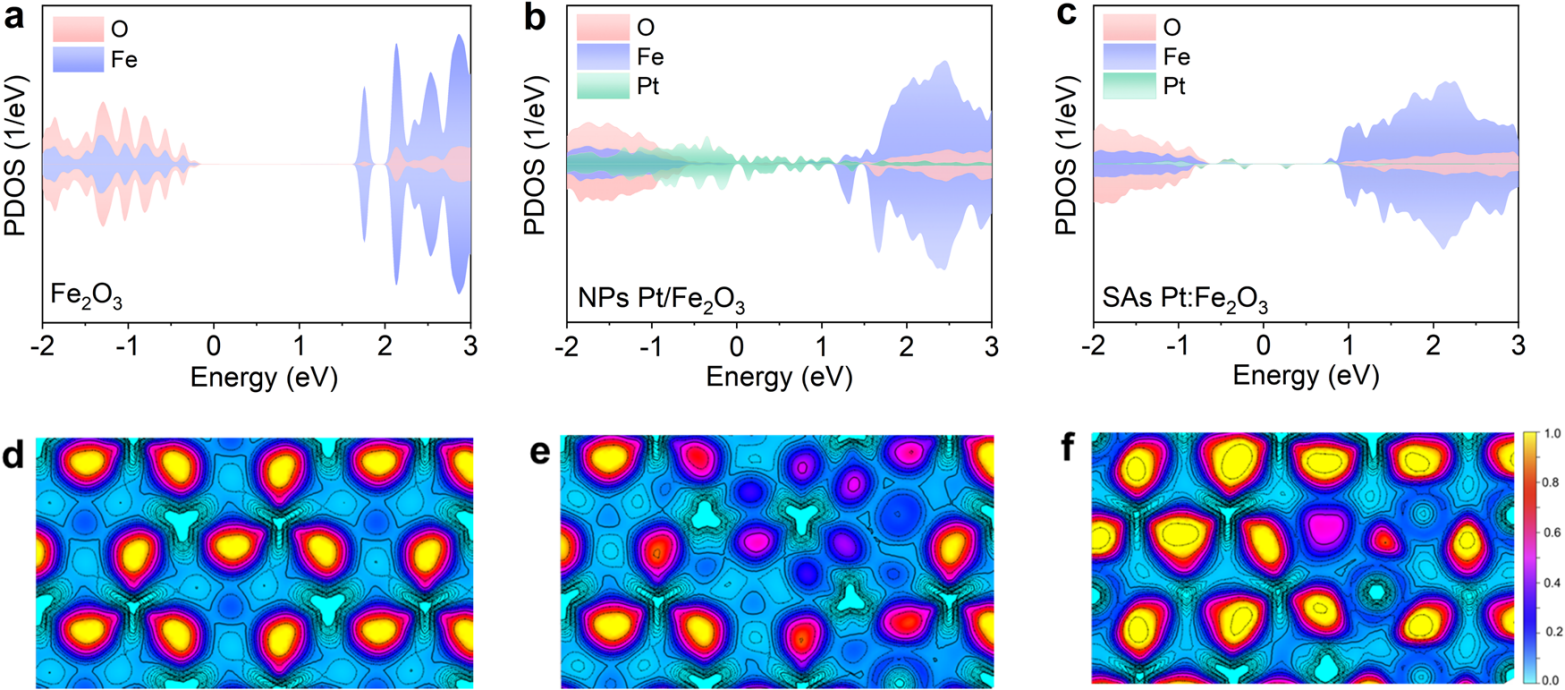
Electronic structures of Fe_2_O_3_, NPs Pt/Fe_2_O_3_, and SAs Pt:Fe_2_O_3_. **a**–**c** Projected density of states (PDOS) states of **a** Fe_2_O_3_, **b** NPs Pt/Fe_2_O_3_, and **c** SAs Pt:Fe_2_O_3_, **d**–**f** electron density distribution plots of **d** Fe_2_O_3_, **e** NPs Pt/Fe_2_O_3_, and **f** SAs Pt:Fe_2_O_3_.

Using the density functional theory (DFT) method, we firstly calculated the projected density of states (PDOS) and electron localization function (ELF) to determine if the loading of Pt nanoparticles (NPs) and single atoms (SAs) can influence the electronic structure and electron mobility of Fe_2_O_3_ geometries, and the samples are labeled as NPs Pt/Fe_2_O_3_ and SAs Pt:Fe_2_O_3_ (Supplementary Fig. 1a–c). The computed direct band gap of Fe_2_O_3_ is approximately 2.0 eV (Fig. 1a), agreeing with the value reported in the literature (2.1 eV)^14,15^. For the NPs Pt/Fe_2_O_3_, partial Pt atoms are bonded to surface oxygens, whilst others exhibit metallic properties, forming the Pt-Pt bonds. The Pt NPs introduce almost continuous states between the band gap of Fe_2_O_3_ (Fig. 1b), promoting the photogenerated electrons transferring from Fe_2_O_3_ to Pt NPs. However, the state created by Pt NPs can act as the charge recombination center. Further downsizing the Pt nanoparticles to single atomic level, Pt atoms form Pt-O covalent bonds with O atoms, which create several charge trapping states within the bandgap compared to that of NPs Pt/Fe_2_O_3_ (Fig. 1c). These trapping states primarily composed of SAs Pt atoms, can trap the photogenerated charge carriers and accelerate the electron-hole separation (discussion in the following). Moreover, ELF shows that doping Pt reduces the electron localization in regard to that of the pristine Fe_2_O_3_ (Fig. 1d–f), benefiting to improve electron transport rate. Comparatively, the strong Pt-O and metal-metal interaction exist in NPs Pt/Fe_2_O_3_ (Fig. 1e), while the latter vanishes in SAs Pt:Fe_2_O_3_ (Fig. 1f). These results confirm that single atomic Pt doping can facilitate the electron separation and transfer in contrast to traditional doping engineering in term of improving performance.

### Synthesis and characterization of SAs Pt:Fe_2_O_3_

**Fig. 2:**
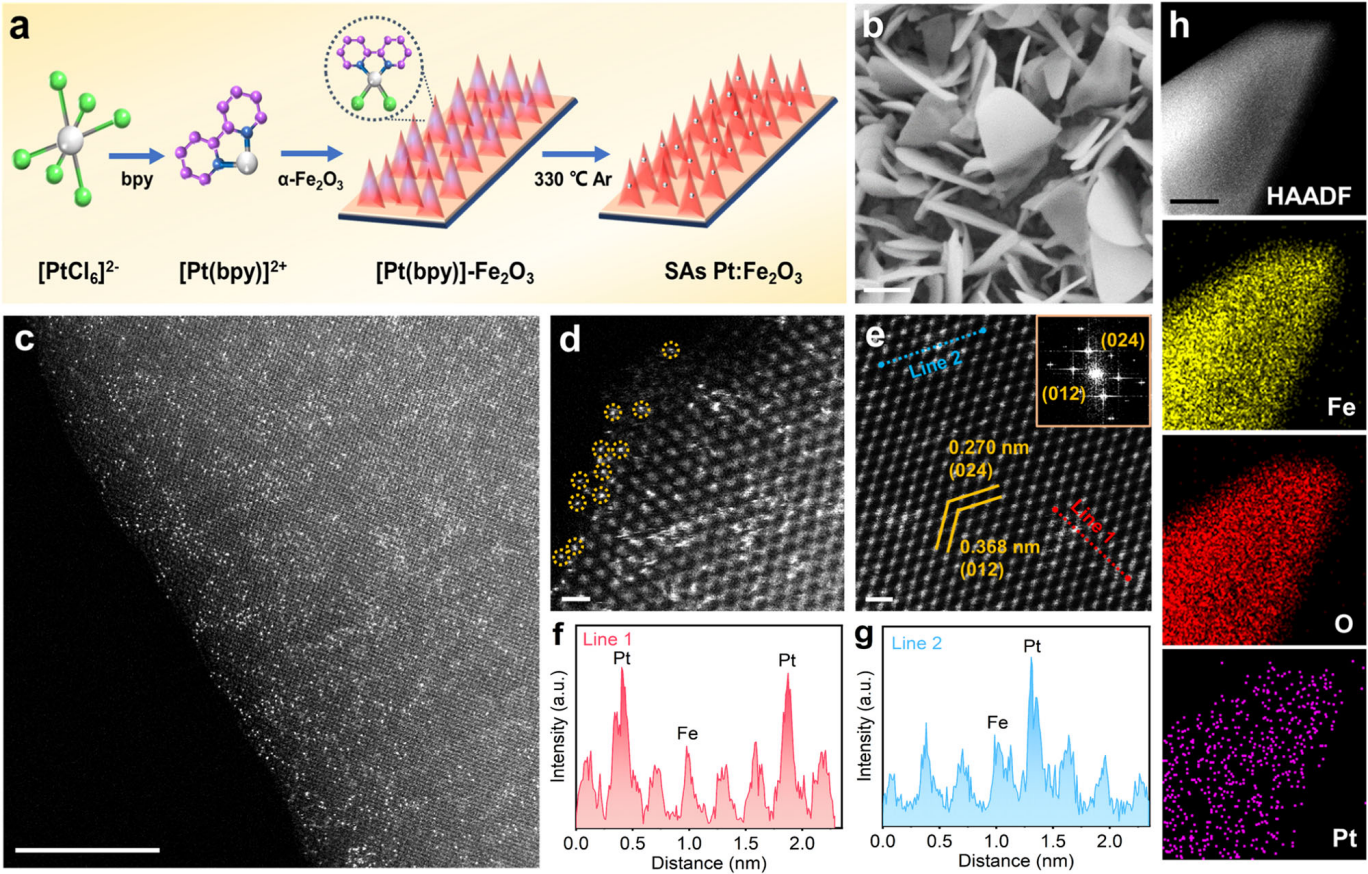
Characterization of SAs Pt:Fe_2_O_3_. **a** Schematic illustration for the synthesis of SAs Pt:Fe_2_O_3_. Color codes: grey (Pt), green (Cl), blue (N), and purple (C); **b** SEM images; **c**–**e** high resolution HAADF STEM images. A large numbers of Pt atoms were atomically dispersed on the Fe_2_O_3_ as observed in **c**. Inset of **e** shows the FFT pattern; **f**, **g** the lines represent the line profiles from HAADF intensity analysis labeled in **e**; **h** elemental mapping. Scale bars in **b**–**e**, and **h** are 200 nm, 5 nm, 500 pm, 500 pm, and 20 nm, respectively.

Motivated by the theoretical calculations, SAs Pt:Fe_2_O_3_ photoanode was synthesized as illustrated in Fig. 2a. To begin with, one-dimensional single-crystalline Fe_2_O_3_ nanoflakes were formed by a thermal treatment at 400 °C for 3 h in air (Supplementary Fig. 2), and subsequently were immersed in the solution of 2,2-bipyridine as the ligand to chelate Pt cations. The ligand was removed under Ar atmosphere at 330 °C to obtain isolated single-atomic Pt doped Fe_2_O_3_ (Supplementary Fig. 3). The NPs Pt/Fe_2_O_3_ was prepared as the thermal temperature was 400 °C (Supplementary Fig. 4). X-ray diffraction (XRD) pattern of SAs Pt:Fe_2_O_3_ displays the Fe_2_O_3_ (JCPDS No. 33-0664), Fe_3_O_4_ (JCPDS No. 19-0629), and Fe peaks (Supplementary Fig. 4d). The formation of Fe_3_O_4_ originated from the thermal annealing treatment owing to the oxygen diffusion from the surface to the bulk material. Fe_3_O_4_ is not photoactive for photoelectrochemical performance, while it can be assured that it is more conducive than the Fe_2_O_3_, and acts as a conductive layer to transfer charge carrier from the Fe_2_O_3_ to the back side^46^. Scanning electron microscopy (SEM) images exhibit one dimensional nanoflakes structure with a length of 1.5–2.5 μm on SAs Pt:Fe_2_O_3_ (Fig. 2b and Supplementary Fig. 5). The aberration-corrected high-angle annular dark-field scanning transmission electron microscopy (AC-HAADF-STEM) images reveal a high degree of single atom dispersion for SAs Pt:Fe_2_O_3_ (Fig. 2c–e and Supplementary Fig. 6), where the Pt atoms are uniformly dispersed at the Fe atom positions (Fig. 2f, g). One can see that the edge of nanoflakes presents the defected structure decorated with the isolated Pt atoms (Fig. 2d and Supplementary Fig. 7). Element mapping shows the homogeneous distribution of Fe, O, and Pt species across the whole nanoflakes (Fig. 2h), indicative of the presence of single atom (Supplementary Fig. 8). Induced coupled plasma-mass spectrometry analysis shows ~4 at.% of Pt content on SAs Pt:Fe_2_O_3_. On the contrary, significant aggregation of Pt species with a size of 5–10 nm can be observed on NPs Pt/Fe_2_O_3_ (Supplementary Figs. 9–11). The controllable experiment using the same manner without 2,2-bipyridine results in the formation of Pt nanoparticles (Supplementary Fig. 12). It should be noted that the use of 2,2-bipyridine and low annealing treatment are the critical factors to realize the single atom sites and maintain the dispersion of Pt species. This is a feasible approach of tailoring Pt single atoms doping into single crystalline photoelectrodes.

**Fig. 3:**
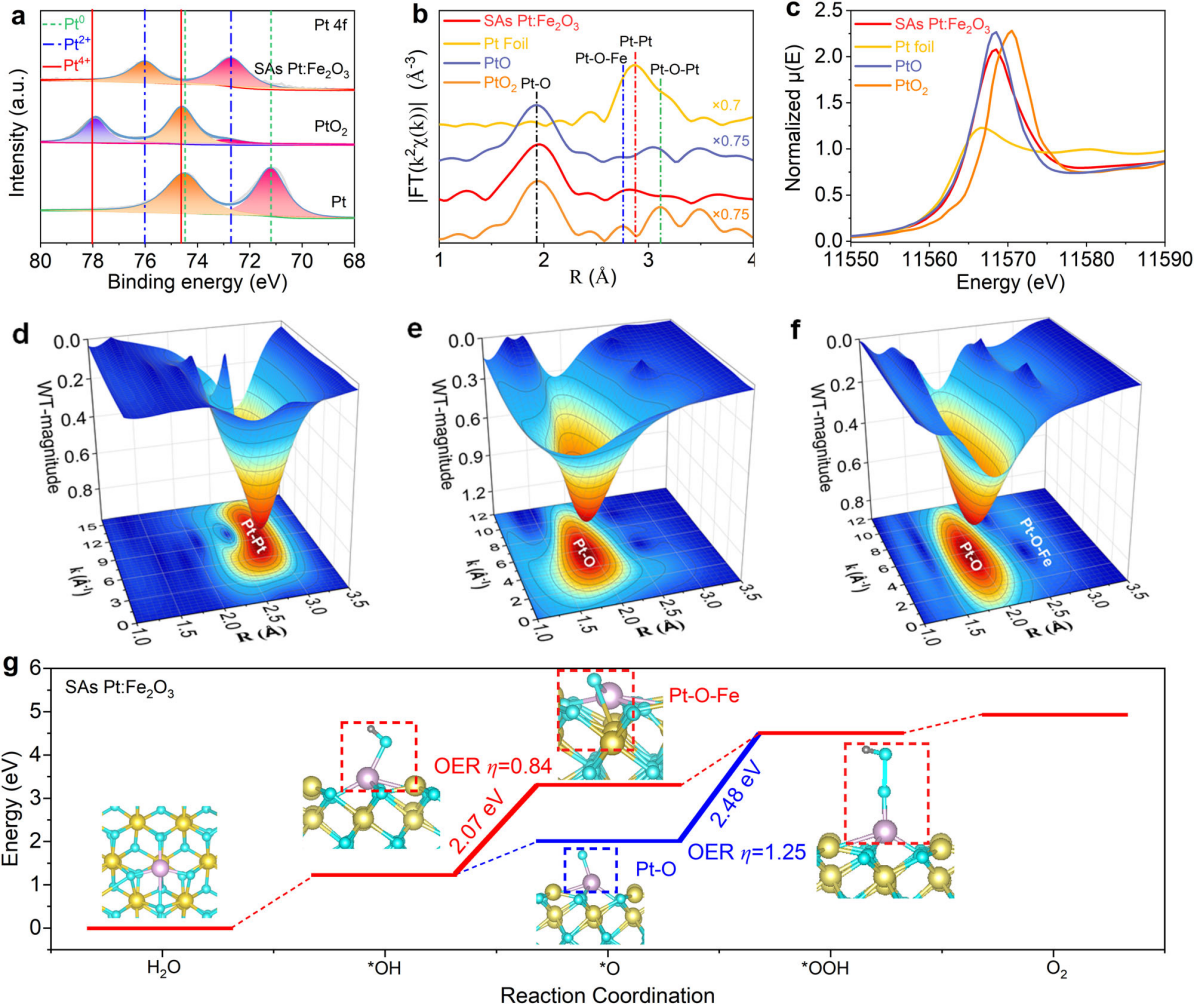
Coordination environment analysis of SAs Pt:Fe_2_O_3_. **a** Pt *4f* XPS spectra; **b** Fourier-transformed *R*-space of the experimental Pt *K*-edge EXAFS signals; **c** normalized XANES spectra; **d**–**f** 3D contour WT-EXAFS maps with 2D projection of **d** Pt foil, **e** PtO, and **f** SAs Pt:Fe_2_O_3_. **g** free energies of OER reaction steps occurring on Pt-O or Pt-O-Fe active sites in SAs Pt:Fe_2_O_3_.

X-ray photoelectron spectra (XPS) and X-ray absorption fine structure (XAS) spectroscopy were used to probe the local coordination chemistry of the Pt species. The binding energies of Pt *4f*_7/2_ (72.80) and Pt *4f*_5/2_ peaks (76.10 eV) on SAs Pt:Fe_2_O_3_ are higher than those of metallic Pt^0^ and lower than those of Pt^4+^ (Fig. 3a), indicating the presence of SAs Pt in hematite. The only Pt *4f* peaks representing the metallic Pt appear in NPs:Fe_2_O_3_ (Supplementary Fig. 13). To further provide the evidence of single atom, Fig. 3b exhibits two peaks at 1.94 Å and 2.84 Å in the Pt *L*-edge X-ray absorption fine structure (EXAFS) spectrum of SAs Pt:Fe_2_O_3_. The first shell originates from the coordination of Pt atom to oxygen (Pt-O), and the second shell fits for the Pt-O-Fe with a comparison between Pt-O-Fe and Pt-Pt coordination models, consistent with the analysis of the dispersed Pt atoms from HADDF-STEM images (Fig. 2c). The contribution of Pt-O and Pt-O-Fe comes from the interaction between single Pt site and oxygen/iron atoms on Fe_2_O_3_. The normalized X-ray absorption near-edge structure (XANES) spectra exhibit the intensity of SAs Pt:Fe_2_O_3_ located at Pt foil and PtO_2_ (Fig. 3c), manifesting that the valence of Pt is lower than +4. The *R* space fitting and EXAFS fitting results suggest that the calculated structure is Pt-O and Pt-O-Fe (Supplementary Fig. 14 and Supplementary Table 2). SAs Pt:Fe_2_O_3_ exhibits Pt-O bonding with coordination number (CN) approaching 4.5 at 1.94 Å, and Pt-O-Fe bonding with CN approaching 1.0 at 2.84 Å in second coordination shell, clearly demonstrating the formation of single atom on hematite. Owing to the powerful resolution in both *k* and *R* spaces, the Pt *L* edge wavelet transform extended EXAFS (WT-EXAFS) was employed to investigate the atomic configuration of SAs Pt:Fe_2_O_3_ with the references of Pt foil and PtO (Fig. 3d–f). For the Pt *L* edge wavelet transform of *χ*(*k*) spectra of SAs Pt:Fe_2_O_3_, one highest merged scattering path signal of Pt-O bond located at [*χ*(*k*), *χ*(*R*)] of [6.0, 2.02] is observed, and a low scattering path signal of Pt-O-Fe bond located at [*χ*(*k*), *χ*(*R*)] of [6.0, 3.0] can be seen (Fig. 3f). The characteristic scattering path signal of Pt-Pt bond in the Pt foil is located at [7.4, 2.80] (Fig. 3d), and it is not detected on SAs Pt:Fe_2_O_3_. The characteristic scattering path signal of Pt-O bonds in the PtO is located at [6.0, 2.0] (Fig. 3e). The WT-EXAFS analysis determines the radial distance and *k* space resolution, confirming the coexistence of Pt-O and Pt-O-Fe without Pt-Pt in SAs Pt:Fe_2_O_3_. The above analyses demonstrate that the dispersed Pt atoms are in the form of Pt-O and Pt-O-Fe bonds in the hematite nanoflakes.

To investigate the real reaction path of four-electron oxygen evolution reaction (OER) in SAs Pt:Fe_2_O_3_, we calculated the thermodynamic free energy diagrams for OER. The side view structures of *OH, *O, and *OOH absorbed on SAs Pt:Fe_2_O_3_ surface are shown in the inset of Fig. 3g. The calculated data in Fig. 3g demonstrate that there are two OER paths (first OER path: Pt-OH → PtOFe → PtOOH → O_2_; second OER path: Pt-OH → PtO → PtOOH → O_2_) in SAs Pt:Fe_2_O_3_ surface. Typically, the higher energy barrier of rate-determining steps, the larger overpotentials (*η*) for OER. As seen in Fig. 3g, we can conclude that the energy barrier of *O intermediate formation in the first OER path (2.07 eV) is lower than that of *OOH intermediate formation in the second OER path (2.48 eV). Thus, the *η* of first OER path (0.84 V) is lower than that of second OER path (1.25 V). The distinct 0.41 V drop in *η* shows that the first OER path is the real reaction path in SAs Pt:Fe_2_O_3_, indicating that Pt-O-Fe is the crucial active site which can promote the surface OER kinetics and improve the solar water splitting activity.

### Photoelectrochemical performance

**Fig. 4:**
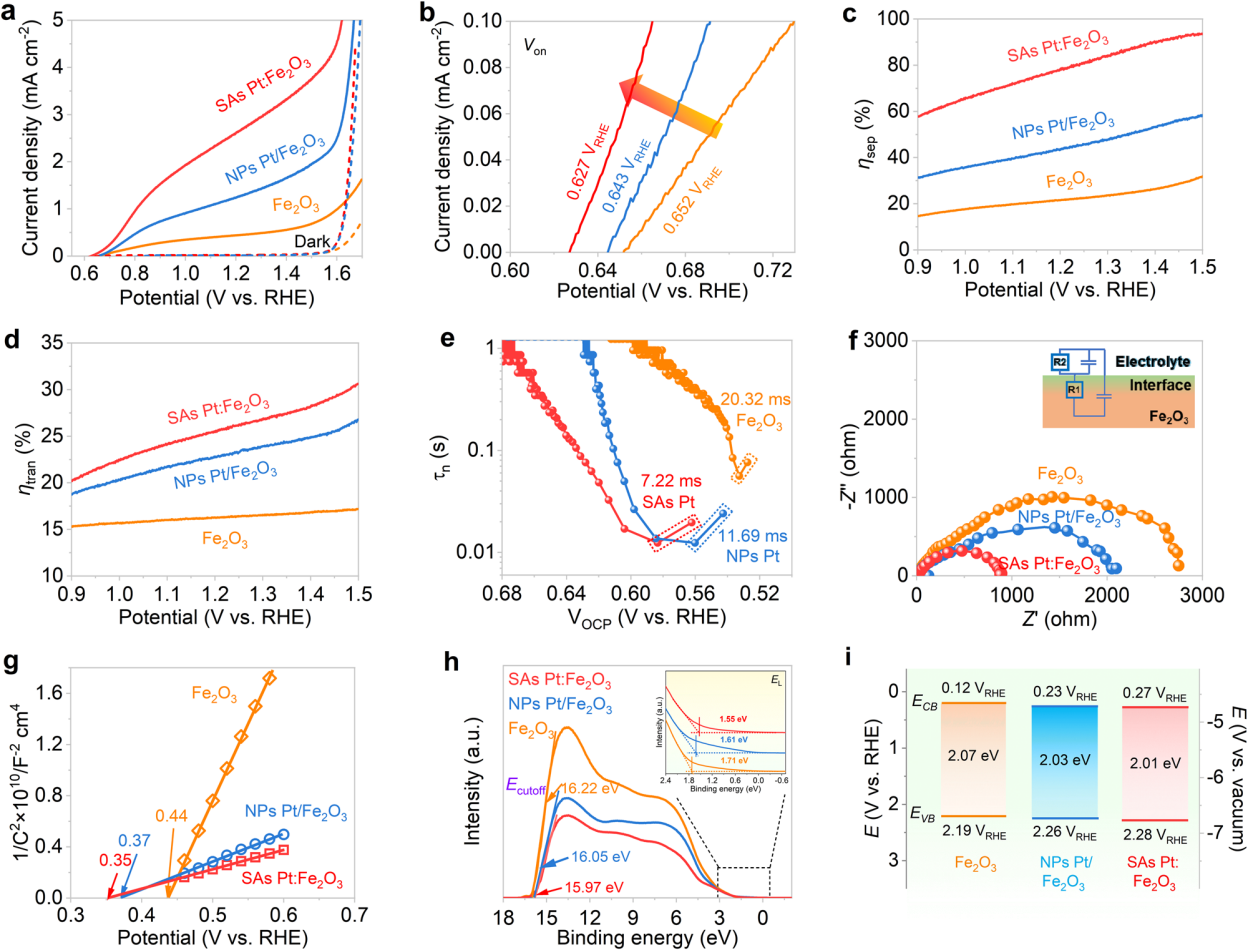
PEC performances and band edge energetics of Fe_2_O_3_, NPs Pt/Fe_2_O_3_, and SAs Pt:Fe_2_O_3_. **a** *J-V* curves; **b** extracted *V*_on_; **c** charge separation efficiencies; **d** charge transfer efficiencies; **e** OCP-derived carrier transfer lifetimes; **f** PEIS at 1.23 V_RHE_. Inset shows the circuit; **g** Mott-Schottky plots; **h** UPS spectra. The black dashed lines indicate the area shown in zoomed-in image in the inset of **h**; **i** band diagrams determined from UV-vis absorption and UPS measurements. All electrochemical measurements were performed in 1 M KOH under AM 1.5G illumination (100 mW cm^−2^).

The PEC performances were investigated in a three-electrode cell (Supplementary Fig. 15). Figure 4a shows the photocurrent-potential (*J-V*) curves of the photoanodes in 1 M KOH under AM 1.5G (100 mW cm^−2^) simulated sunlight. The pristine Fe_2_O_3_ displays a photocurrent density of 0.45 mA cm^−2^ at 1.23 V_RHE_ with an onset potential (*V*_on_) of 0.652 V_RHE_. With the introduction of Pt dopant into hematite, both NPs and SAs enhance the photocurrent densities of hematite, accompanied by the negative shift of *V*_on_, indicating the fast charge transfer by Pt incorporation. Dark current densities for both samples are also remarkably improved with a cathodic shift of approximately 58 mV at 0.5 mA cm^−2^ (Supplementary Fig. 16). The optimum SAs Pt:Fe_2_O_3_ shows a photocurrent density of 2.71 mA cm^−2^ at 1.23 V_RHE_ (Supplementary Fig. 17), higher than that of NPs Pt/Fe_2_O_3_ (1.29 mA cm^−2^). Moreover, the *V*_on_ of SAs Pt:Fe_2_O_3_ (0.627 V_RHE_) is found to be negatively shifted by 25 and 16 mV relative to those of the pristine Fe_2_O_3_ and NPs Pt/Fe_2_O_3_ (Fig. 4b), suggesting the enhanced surface water oxidation kinetics, especially the surface edge with the substitution of single atomic Pt (Fig. 2d). The improved photocurrent and the reduced *V*_on_ increase ABPE with a maximum value of 0.51% (Supplementary Fig. 18), which surpasses the previously reported the highest value of 0.31%^27^. Besides, the current spike on SAs Pt:Fe_2_O_3_ (*i*/*i*_0_ = 0.91) is reduced from the chopped *J-V* plots (Supplementary Fig. 19), indicating promoting sluggish water oxidation by single atomic Pt.

To understand the charge character by the influence of SAs Pt, charge separation efficiency (*ƞ*_sep_) and charge transfer efficiency (*ƞ*_tran_) were calculated, and derived from the *J-V* curves measured in a hole scavenger-containing electrolyte (Supplementary Fig. 20). The *ƞ*_sep_ points to the hole extraction from the excited Fe_2_O_3_ to the surface. From Fig. 4c, Pt doping dramatically improves the *ƞ*_sep_ in the entire voltage region. SAs Pt:Fe_2_O_3_ presents higher *ƞ*_sep_ values in relative to the traditional particles doping, reaching to 80.1% at 1.23 V_RHE_, while NPs Pt/Fe_2_O_3_ arrives to 44.1% of the same potential. Single atom edging at the surface also plays a vital role for the hole trapping to the surface for water oxidation. Besides, SAs Pt:Fe_2_O_3_ exhibits a *ƞ*_tran_ value of 25.8% at 1.23 V_RHE_ (Fig. 4d), higher than that of NPs Pt/Fe_2_O_3_ (23.0%), indicating that the fraction of holes reaches to the semiconductor/electrolyte interface without significant recombination in the bulk material. All these results confirm that adjusting the coordination environment via atom-level regulation for PEC water splitting evidently promotes to the charge separation/transfer and realizes efficient energy conversion.

Open circuit potential (OCP) transient decay profile provides an additional information on the photogenerated charge carrier behavior. The OCP in dark is positive and then shifts to the negative direction under illumination (Supplementary Fig. 21a), which is caused by built-in electric field generated by the photo-generated carriers^27^. SAs Pt:Fe_2_O_3_ presents a strikingly accelerated OCP decay as terminating the illumination in relative to NPs Pt/Fe_2_O_3_, representing a large photovoltage generation (*Δ*OCP = OCP_dark_ - OCP_light_). Charge transfer and recombination are principally two competing processes that determine the water oxidation rate on the photoanode surface. To further clarify the charge recombination rate at the semiconductor/electrolyte junction, the carrier transfer lifetime was calculated from the derived-OCP values, as depicted in Fig. 4e (see details for supporting information). NPs Pt/Fe_2_O_3_ displays a carrier lifetime of 11.69 ms when illumination was removed, one time lower than the pristine Fe_2_O_3_ (20.32 ms). SAs Pt substitution continues to reduce upon 7.22 ms on Fe_2_O_3_, indicating effective charge separation and fast transfer kinetics. In addition, the incident photon-to-current conversion efficiency (IPCE) on SAs Pt:Fe_2_O_3_ is remarkably improved over the entire range of 340-600 nm (Supplementary Fig. 21b).

Figure 4f shows the photoelectrochemical impedance spectroscopy (PEIS) for the corresponding photoanodes, and the equivalent circuit was employed to fit the Nyquist plots of PEIS (inset of Fig. 4f). SAs Pt:Fe_2_O_3_ exhibits a considerably lower resistance (546.50 Ω) than NPs Pt/Fe_2_O_3_ (809.80 Ω) and Fe_2_O_3_ (2719.40 Ω), as summarized in Supplementary Table 3. And, SAs Pt:Fe_2_O_3_ remains a relatively stable photocurrent for 10 h durability (Supplementary Fig. 22a). The gases produced from the working and counter electrodes present the evolved O_2_ and H_2_ with a ratio of 2:1 with the Faraday efficiencies of gas productions closed to 100% (Supplementary Fig. 22b). In fact, anion and cations species modulation allow to induce extra electrons near Fe^3+^ sites to form Fe^2+^ sites, which can enhance the electrical conductivity of Fe_2_O_3_. However, high amount metal doping would principally induce the recombination centers by creating inter-bandgap energy states since the Fe^2+^ sites are near to the surface region, causing trapping states and high overpotential for water oxidation. The coordination of single atomic-level Pt substitution for Fe^3+^ can avoid the high concentration of doping to a certain degree, which increases the polaron hopping probability, and feasibly enhances the photogenerated charge carrier rate for PEC reaction, consistent with the DFT calculation. On the other hand, atom-level substitution near to the surface would inhibit the interfacial charge recombination, which is reflected by the low *V*_on_.

The flat band potentials (*E*_fb_) of SAs Pt:Fe_2_O_3_ and NPs Pt/Fe_2_O_3_ were conducted by Mott-Schottky measurement. *E*_fb_ is cathodically shifted since Pt was induced (Fig. 4g), coincided with the shift of *V*_on_ from the *J-V* plot (Fig. 4b). SAs Pt:Fe_2_O_3_ has shown a lower *E*_fb_ (0.35 V_RHE_) than NPs Pt/Fe_2_O_3_ (0.37 V_RHE_), manifesting the change in the band energetics. Besides, the similar slopes of Mott–Schottky plots represent the resembled carrier concentration, implying the close donor densities in the supporting materials loading with Pt nanoparticles and single-atom. Ultraviolet photoelectron spectroscopy (UPS) and ultraviolet-visible (UV-Vis) absorption were implemented to illustrate the band edge positions of the photoanodes. Tauc plots of photoanodes declare the band gaps of the pristine Fe_2_O_3_, NPs Pt/Fe_2_O_3_, and SAs Pt:Fe_2_O_3_ are 2.07, 2.03, and 2.01 eV (Supplementary Fig. 23), respectively, coherent with the decreased slopes of Mott-Schottky (Fig. 4g). The work functions of the samples were determined by subtracting the cutoff energies (*E*_cutoff_) from the UPS curves (Fig. 4h). The positions of the valence band maxima in regard to the Fermi levels were derived from the onset of valence band photoemission on the low binding energy edges of UPS spectra (inset of Fig. 4h). Based on these results, the determined band positions for the corresponding samples are summarized in Fig. 4i and Supplementary Table 4, according to the literatures reported^3,47^. One can see that tailoring Pt-O sites can influence the band structure, promote the charge separation, and accelerate the photogenerated holes transferring to the semiconductor/electrolyte surface for water oxidation, therefore enhancing PEC performance.

### IMPS and TAS analysis for carrier kinetics

**Fig. 5:**
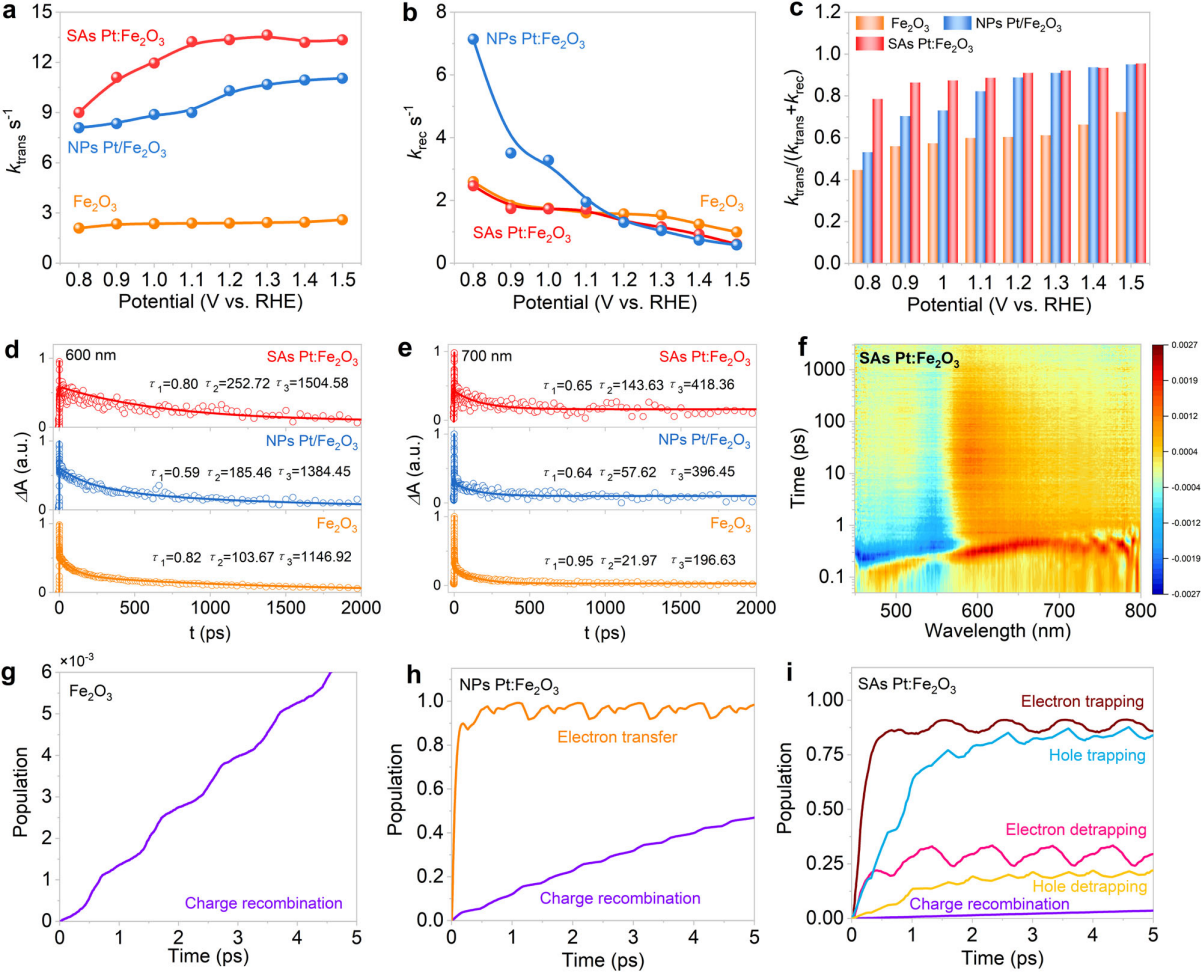
IMPS and TAS for Fe_2_O_3_, NPs Pt/Fe_2_O_3_, and SAs Pt:Fe_2_O_3_. **a** Charge transfer rate constant (*k*_trans_); **b** charge recombination rate constant (*k*_rec_); **c** charge transfer efficiencies extracted from IMPS analysis; **d**, **e** transient absorption decays observed at **d** 600 nm and **e** 700 nm. The fits for the decays were calculated with three exponential decay model, shown as the solid lines, and the circles represent experimental data; **f** time-resolved transient absorption spectra of SAs Pt:Fe_2_O_3_ when excited with 380 nm; **g**–**i** electron trapping, electron detrapping, hole trapping, and charge recombination of **g** Fe_2_O_3_, **h** NPs Pt/Fe_2_O_3_, and **i** SAs Pt:Fe_2_O_3_.

Furthermore, we performed intensity modulated photocurrent spectroscopy (IMPS) and transient absorption spectroscopy (TAS) to understand the charge carrier kinetics in various Fe_2_O_3_ photoanodes. The semicircle coincides with the competition between interfacial carrier transfer and electron-hole recombination (Supplementary Fig. 24). The frequency of the maximum imaginary is associated with the sum of charge transfer (*k*_trans_) and recombination (*k*_rec_) rate constants (*k*_trans_+*k*_rec_)^48–50^. The ratio of *k*_trans_/(*k*_trans_+*k*_rec_) is taken by comparing the instantaneous photocurrent and steady-state photocurrent. SAs Pt:Fe_2_O_3_ exhibits the highest *k*_trans_ values over the measured potential range (Fig. 5a), indicating that single atoms speed up the charge carrier mobility and quickly transfer from the bulk material to the surface or to the back contact. By contrast, the *k*_rec_ of SAs Pt:Fe_2_O_3_ is lower than that of NPs Pt/Fe_2_O_3_, and almost overlapped with that of the pristine Fe_2_O_3_ in the potential of 0.8–1.1 V_RHE_ (Fig. 5b), indicating high charge transfer rate of SAs Pt:Fe_2_O_3_. Further considering surface substitution by SAs Pt, Fermi level pinning effect caused by surface states can be eliminated. With the Pt nanoparticles decorated on the surface, the aggregated particles are inefficient to change the inherent surface trap states possibly owing to the deep-level defects, while atomic Pt substitution does not induce more defects in hematite (Supplementary Fig. 25). Further on, the high charge transfer efficiency of *k*_trans_/(*k*_trans_+*k*_rec_) can be obtained on SAs Pt:Fe_2_O_3_, especially in the potential range of 0.8-1.1 V_RHE_ (Fig. 5c), suggesting that the charge recombination is greatly reduced via single atomic bulk and surface doping. In other words, Pt-O coordination favors the photoresponse at low potentials.

TAS can be used to characterize the dynamics of photoinduced charge carrier. The distinct absorption spectra of photoelectrons and holes in photoanode make it possible to monitor the concentration variation of the species, especially the fates of photoholes and photoelectrons on the timescale of picosecond to microseconds. TAS of hematite principally presents two bands, where a bleach band at a probe wavelength of ~600 nm is ascribed to trapping photoelectrons, and an absorption band of ~700 nm corresponds to photoholes^51,52^. Under OCP condition (Supplementary Fig. 26), the photogenerated charge carrier dynamics can be associated to the bulk electron-hole recombination since the decay dynamics are faster than the timescale of water oxidation on the surface. From the decay dynamics photoinduced absorption (Fig. 5d–f), the carrier dynamics for the samples was calculated by fitting the kinetics traces at 600 nm and 700 nm (Supplementary Table 5). The charge carrier decay average lifetime of *τ*_av_ at 600 nm extends from 304.49 ps (Fe_2_O_3_) to 437.28 ps for NPs Pt/Fe_2_O_3_ and 486.76 ps for SAs Pt:Fe_2_O_3_. The increased charge carrier decay lifetime of the absorption signal indicates that single atom doping can effectively promote charge separation, extend charge carrier decay lifetime, and reduce the charge recombination on the picosecond time scale. SAs Pt:Fe_2_O_3_ shows the long lifetimes at 600 and 700 nm relative to NPs Pt/Fe_2_O_3_ and Fe_2_O_3_, indicative of the long-lived photoholes for water oxidation. The pristine Fe_2_O_3_ exhibits a fast decay among all samples due to the serious electron-hole recombination. These provide strong evidence that the charge recombination kinetics can be greatly suppressed by SAs Pt doping, which favors to the water photo-oxidation of hematite under the present experimental conditions.

We next carried out the nonadiabatic (NA) molecular dynamics with decoherence-induced surface hopping (DISH) approach to analysis the charge carrier dynamics of hematite, and the simulated data are shown in Fig. 5g–i. Fitting the curves to an exponent, $P\left(t\right)=\exp (-\frac{t}{\tau })$, we obtained the charge trapping, detrapping, transfer, and recombination time scales (Supplementary Table 6). In general, electron-hole recombination results in short carrier lifetime and reduces OER performance. In the pristine Fe_2_O_3_ system, the photogeneration charge carriers occur the detrimental recombination process (Fig. 5g). Doping the Fe_2_O_3_ with Pt NPs (Fig. 5h), free carrier recombination process is inhibited compared to the Fe_2_O_3_ system, and the electron transfer between Fe_2_O_3_ and NPs Pt becomes the main process. The fast charge transfer promotes efficient charge separation and prolongs the carrier lifetime. Changing the NPs Pt to SAs Pt (Fig. 5i), the occurrence of free carrier recombination is further reduced compared to NPs Pt/Fe_2_O_3_ system, and the charge carriers mainly take place in trapping and detrapping processes. The charge trapping and detrapping are in dynamic equilibrium, which extends the carrier lifetime and improves the OER performance of Fe_2_O_3_ comparable to NPs Pt doping, demonstrating the above experimental results.

### Further PEC performance enhancement via surface oxygen vacancies

**Fig. 6:**
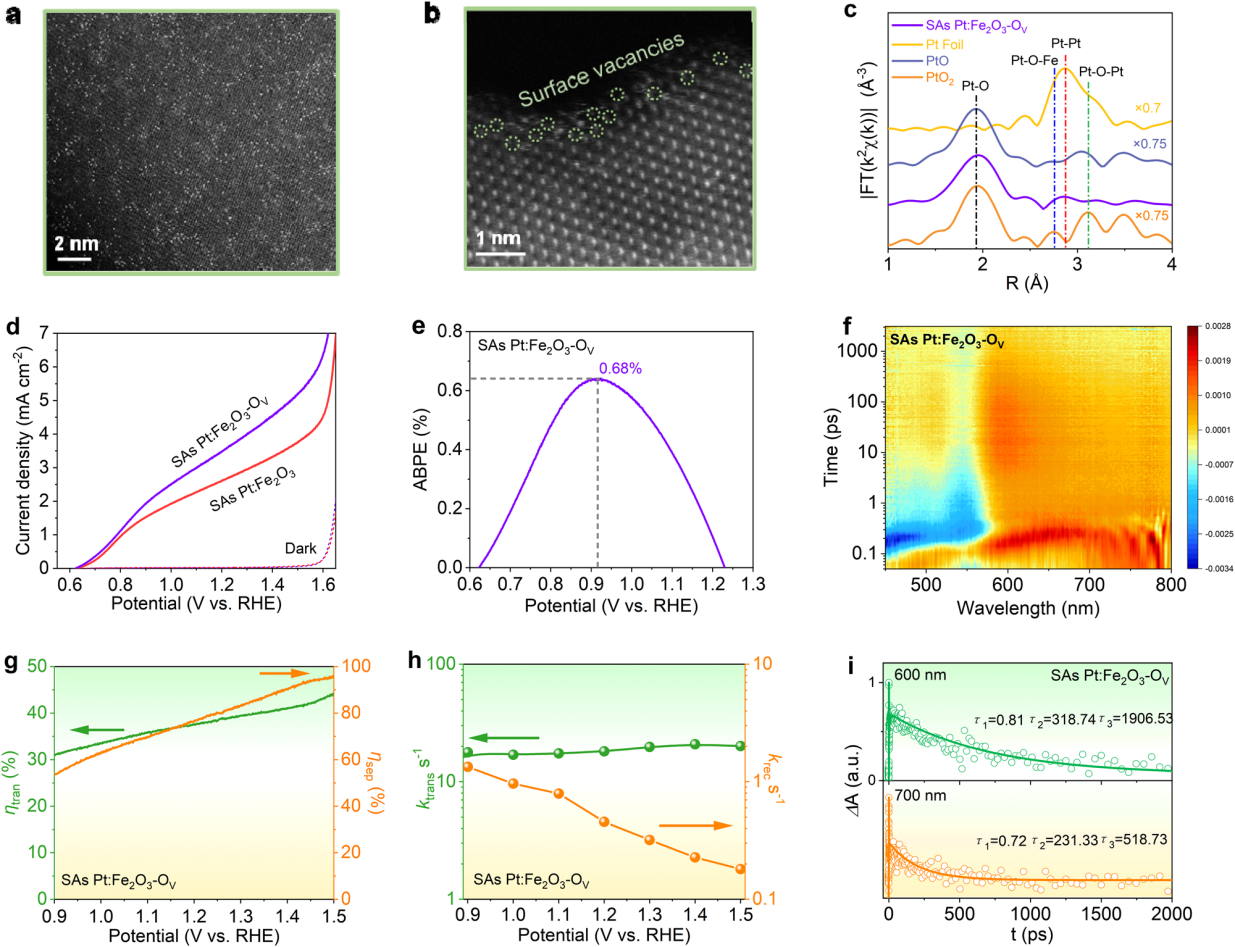
Performance improvement of SAs Pt:Fe_2_O_3_-Ov. **a**, **b** High resolution HAADF STEM images; **c** *k*3-weighted Fourier-transform spectra from EXAFS; **d** *J-V* curves in 1 M KOH under AM 1.5G illumination; **e** ABPE value; **f** time-resolved transient absorption spectra when excited with 380 nm; **g** charge separation and charge transfer efficiencies; **h** charge transfer rate constants (*k*_trans_)and charge recombination rate constants (*k*_rec_); **i** transient absorption decays observed at 600 and 700 nm. The fits for the decays were calculated with three exponential decay model, shown as the solid lines, whereas the circles represent experimental data.

Benefited from the role of single atom substitution, we further used plasma etching treatment to produce surface oxygen vacancies (O_V_) and accelerate the charge transfer on SAs Pt:Fe_2_O_3_ (remarked as SAs Pt:Fe_2_O_3_-O_V_). No evident morphology change can be discerned for the treated sample (Supplementary Fig. 27). Single atoms are still retained from HAADF-STEM observation (Fig. 6a). Surface vacancies can be viewed as marked by the circle (Fig. 6b), accompanied by the irregular surface boundary originated from plasma treatment (Supplementary Fig. 28). Moreover, SAs Pt:Fe_2_O_3_-Ov maintains two peaks at 1.96 Å and 2.86 Å in the Pt *L*-edge EXAFS spectrum (Fig. 6c), representing the coordination of Pt-O and Pt-O-Fe (Supplementary Fig. 29). The slight decrease in peak intensity on SAs Pt:Fe_2_O_3_-O_V_ can be observed compared with SAs Pt:Fe_2_O_3_ (Supplementary Fig. 30), reflecting a decrease in the coordination number of Pt to O ascribed to the presence of oxygen vacancies. The SAs Pt:Fe_2_O_3_-O_V_ displays Pt-O bonding with CN approaching to 4 at 1.96 Å in the first coordination shell (lower than SAs Pt:Fe_2_O_3_ with CN of 4.5), while Pt-O-Fe bonding with CN approaching 1 at 2.86 Å in the second coordination shell (Supplementary Table 7). The fitting results of *χ*(*R*) and *χ*(*k*) space spectra with reasonable *R*-factor quantitatively support the local atomic structure and coordination numbers information. O *1s* XPS spectrum shows that the lattice oxygen is shifted to higher binding energy by 180 mV for SAs Pt:Fe_2_O_3_-O_V_, while Pt *4f* XPS spectrum is negatively shifted after plasma treatment (Supplementary Fig. 31). This means that SAs Pt in Fe_2_O_3_-O_V_ are electron-richer compared with SAs Pt:Fe_2_O_3_, and more charge can be transferred from Fe_2_O_3_ to Pt. Additionally, Pt content on the top surface has decreased from 9.96 (without plasma treatment) to 6.97 at.% (Supplementary Table 8). This can be originated from the generated surface O_V_, which anchors the Pt single atom to stabilize it.

The PEC performance of SAs Pt:Fe_2_O_3_-O_V_ is further improved after optimization (Supplementary Fig. S32), in which the photocurrents reach to 3.65 and 5.30 mA cm^-2^ at 1.23 and 1.5 V_RHE_ with a maximum ABPE value of 0.68% (Fig. 6d, e). This is more than double the previously reported highest value of the doped Fe_2_O_3_-based photoanodes (Supplementary Table 1), and even superior to the cocatalysts decorated Fe_2_O_3_-based photoanodes (Supplementary Table 9). Meanwhile, *V*_on_ shifts to the negative direction with ∆*V*_on_ = 5 mV, in line with the negative shift of the flat band potential on SAs Pt:Fe_2_O_3_-O_V_ deduced from the Mott–Schottky plot (Supplementary Fig. 33). This is currently known as one of the best performances in terms of photocurrent and *V*_on_ for elemental doping hematite semiconductors. Moreover, the electrochemical specific surface area of SAs Pt:Fe_2_O_3_-O_V_ is evidently increased, almost 4 times higher than that of the SAs Pt:Fe_2_O_3_ (Supplementary Fig. 34). There is no notable enhancement on the charge separation between SAs Pt:Fe_2_O_3_-O_V_ and SAs Pt:Fe_2_O_3_, but the charge transfer efficiency of SAs Pt:Fe_2_O_3_-O_V_ highly increases (Fig. 6g and Supplementary Fig. 35), supporting the charge transfer influenced by the oxygen vacancies. The charge carrier derived from OCP displays that SAs Pt:Fe_2_O_3_-O_V_ has faster photoresponse (Supplementary Fig. S36), along with higher *k*_trans_ value, lower *k*_rec_ values (Fig. 6h and Supplementary Fig. 37), and long charge carrier lifetime (Fig. 6f, i) comparable with non-treated SAs Pt:Fe_2_O_3_ (Supplementary Fig. 38). The surface recombination is generally happened which affects the oxygen evolution reaction and PEC activity of the photoelectrode. Here the surface oxygen vacancies induced can serve as surface states below the conduction band minimum that boost the charge carrier transfer. The defects raise the flat band potential away from the redox potential of O_2_/H_2_O and increase the upward bending of the hematite band edge. This can facilitate the charge transfer from the Fe_2_O_3_ surface to the electrolyte. In other words, surface oxygen vacancies are conductive to a reduced interfacial charge-transfer barrier and surface trapping states. PDOS of SAs Pt:Fe_2_O_3_-O_V_ demonstrates that oxygen vacancies can eliminate the trap states between the band gap of Fe_2_O_3_ (Supplementary Fig. 39a, b), reducing the charge recombination. Also, ELF (Supplementary Fig. 39c) shows that oxygen vacancies noticeably reduce electron localization and electron transport resistance, which act as active sites for chemisorption of the intermediates, and improve the surface water ability. All these results demonstrate that the synergistic effect of single atom coordination and surface oxygen vacancies are responsible for the improved charge transfer and the suppressed photogenerated charge recombination, boosting water oxidation ability.

Based on the calculation and experimental results above, a mechanism is proposed to clarify the improved charge carrier kinetics for SAs Pt decorated on the defected Fe_2_O_3_. The influence of charge transfer and recombination is schematically illustrated in Supplementary Fig. 40. When Fe_2_O_3_ is exposed to light to generate photo-generated carriers, most of the photo-generated carriers would be recombined instantaneously before arriving to the surface for water oxidation reaction owing to the short hole diffusion length. Few separated photogenerated electrons and holes in the conduction band and the valence band are transferred to the corresponding surface to participate in oxygen and hydrogen evolution reactions, causing the poor performance. When Pt species were incorporated to Fe_2_O_3_, the mobility of excited carrier can be accelerated, thereby improving the carrier transfer efficiency. In contrast to the traditional element doping, single atoms coordination is in favor of improving the charge separation efficiency and prolonging the charge carrier lifetime by the shift of the photogenerated holes towards the photoanode/electrolyte interface and of the electrons to the back side. On the other hand, single atom-level substitution can efficiently suppress the deep-level defects in hematite relative to the nanoparticle/cluster-level doping, in which the latter with aggregated defects has a distribution along the photoanode. Further surface oxygen vacancies were produced in the material, thereby surface oxygen vacancies and reduced Fe^2+^ species within the nanoflakes following the doping reaction mechanism. In addition, one-dimensional single-crystalline nanoflakes structure with enough light harvesting could facilitate the charge collection efficiency.

## Discussion

In summary, single platinum atom doped into Fe_2_O_3_ photoanode has been successfully synthesized by using 2,2-bipyridine as the ligand to chelate Pt cations, followed by the inert atmosphere treatment. Compared with NPs Pt loading, SAs Pt:Fe_2_O_3_ can obviously suppress the deep-level defects and shift the band edge positions of Fe_2_O_3_. The introduction of surface oxygen vacancies further enhances the PEC activity of the SAs Pt:Fe_2_O_3_. The resulting photoanode achieves the photocurrent densities of 3.65 and 5.30 mA cm^−2^ at 1.23 and 1.5 V_RHE_ with a high ABPE of 0.68% for the hematite-based photoanodes. The synergism of single atomic-level doping and surface oxygen vacancies improve charge carrier separation capability and injection efficiency at the semiconductor/electrolyte interface, prolong the charge carrier lifetime, and promote the reaction kinetics. These results clarify the importance of the designing single atom doping engineering in the photoelectrode to boost the efficient charge transfer and extending charge lifetime, and provide a design idea to construct highly efficient photoelectrodes for improving the solar conversion efficiency of other semiconductors.

## Methods

### Material preparation

Iron foils (Alfa Aesar, 0.25 mm thick, 99.99%) with a size of 1 cm^2^ were degreased by ultrasonic treatment in acetone and ethanol for 10 min, and then dried in a nitrogen stream. To prepare the α-Fe_2_O_3_ nanoflakes grown on iron substrate, iron foils were thermally annealed in a furnace (HF-Kejing Furnace, KSL-1100X) at 400 °C in air at a heating rate of 10 °C min^−1^ and kept at the required temperature for 3~4 h. The entire sample surface became nanoflakes during the thermal annealing for the following treatment. Hexachloroplatinic acid hexahydrate (5 mmol) dissolved in ethanol was mixed with 2,2-bipyridine in a molar ratio of 1:1, 1:3, and 1:6 for 10 min The α-Fe_2_O_3_ nanoflakes were immersed into the above solution for different times (5 min, 15 min, 25 min, and 35 min), following by drying overnight in a vacuum oven at 60 °C. Next, the samples were annealing in Ar at 330 °C and 400 °C for 100 min for the synthesis of SAs Pt:Fe_2_O_3_ and NPs Pt/Fe_2_O_3_, respectively. For the SAs Pt:Fe_2_O_3_-O_V_, SAs Pt:Fe_2_O_3_ was treated in a plasma etching condition (HF-Kejing, PEC-500 W) in Ar at 20 W for various times (5 min, 15 min, 25 min, and 35 min).

### Photoelectrochemical characterization

Photoelectrochemical measurements were measured in a standard three-electrode system with a CHI 760D electrochemical analyzer. The light source was used the simulated AM 1.5 G (100 mW cm^−2^) sunlight. The solar simulator used for PEC measurement was equipped with a total-reflection mirror and AM 1.5G fitter, and the spectrum was measured as shown in Supplementary Fig. 41. 1 M Potassium hydroxide (KOH, pH = 14) was used as an electrolyte. The prepared sample, Pt foil, and Ag/AgCl were used as the working electrode, counter electrode, and reference electrode, respectively. Photocurrent vs voltage (*I-V*) curves were recorded by scanning the potential from −0.5 to 0.65 V_Ag/AgCl_ with a rate of 10 mV s^−1^. The measured potential was converted into a potential with respect to a reversible hydrogen electrode (RHE). There is no iR correction performed in the experiment. Electrochemical impedance spectroscopy (EIS) was performed at 1.23 V_RHE_ and a small AC amplitude of 10 mV in the frequency range of 10^−2^–10^5^ Hz under AM 1.5 G illumination.

Applied bias photon-to-current efficiency (ABPE) can be calculated using the following equation of *ABPE*(%) = $\frac{J\times \left(1.23-{V}_{b}\right)}{{p}_{{total}}}$, in which *J* is the photocurrent density (mA cm^−2^) obtained from AM 1.5G illumination, *V*_*b*_ refers to the applied bias potential versus RHE, and *P*_*total*_ is the total light intensity of AM 1.5 G. The incident photoelectron conversion efficiency (IPCE) was measured in 1 M KOH at a potential of 1.23 V_RHE_ in a Xe lamp. IPCE was calculated using the formula of *IPCE*(%) = $\frac{J\times 1240}{\lambda \times {p}_{{Light}}}\times 100\%$, where *J* presents the photocurrent density (mA cm^−2^), *λ* and *P*_light_ are the incident light wavelength (nm) and the power density obtained at a specific wavelength (mW cm^−2^), respectively. Charge separation efficiency (*η*_*sep*_, the yield of photo-generated holes reaching the semiconductor/electrolyte interface) and surface charge transfer efficiency (*η*_*trans*_, the yield of holes participating in the water oxidation reaction after reaching the electrode/electrolyte interface) can be calculated using the following formula of *η*_*sep*_ = $\frac{{J}_{{H}_{2}O}}{{J}_{H2O2}}$ and *η*_trans_ = $\frac{{J}_{H2O2}}{12.5}$, in which *J*_*H2O*_ and *J*_*H2O2*_ are the photocurrent densities obtained in 1 M KOH without and with H_2_O_2_, 12.5 mA cm^−2^ is the theoretical photocurrent of Fe_2_O_3_ under AM 1.5 G illumination. For comparison of charge recombination rate at the photoanode/electrolyte junction, the carrier lifetime was quantified by *τ*_n_ (*τ*_n_=$\frac{{K}_{B}}{e}({\frac{{dOCP}}{{dt}}})^{-1}$).

### Material characterization

The crystalline structure and composition were performed by X-ray diffraction analysis (XRD, Rigaku RINT-2000, Cu *K*α radiation at 40 kV and 40 mA) and X-ray photoelectron spectroscopy (XPS, ESCALAB 250xi, Thermo Fisher Scientific). The elemental contents were tested by inductively coupled plasma optical emission spectroscopy (ICP-OES-720ES, Agilent, USA). The morphology was observed using field emission scanning electron microscopy (FE-SEM, Supra 55, Zeiss, Germany) and transmission electron microscopy (TEM, JEM-2100F, JEOL, Japan) systems. The cross-sectional SEM image was taken by argon-ion milling machine (GATAN, ILION693) with a voltage of 5 kV, following with SEM observation. The aberration-corrected high-angle annular dark-field scanning transmission electron microscopy was performed using JEM-ARM200F. UV-visible diffuse reflectance spectra were implemented on a UV-2600 (Shimadzu) spectrometer using BaSO_4_ as the reference. Photoluminescence (PL) spectra were performed on a HORIBA Fluoromax-4 (HORIBA JY, HORIBA Fluoromax-4, USA) under laser excitation at 350 nm. The electron paramagnetic resonance (EPR) measurements were recorded using a JES-FA200 spectrometer at low temperature (−150 °C).

### Charge carrier kinetics measurements

Intensity modulated photocurrent spectroscopy (IMPS) was performed with a potentiostat (PGSTAT302N, Metrohm), an impedance analyser (FRA32M, Metrohm), and a light-emitting diode (LED) driver kit (Metrohm) that drove illumination of 420 nm power UV LED in 1 M KOH at different voltages (0.8 V_RHE_, 0.9 V_RHE_, 1.0 V_RHE_, 1.1 V_RHE_, 1.2 V_RHE_, 1.3 V_RHE_, 1.4 V_RHE_, and 1.5 V_RHE_). The LED intensity was 5.5 mW cm^−2^, and it was modulated by 10% in the range of 10 kHz–0.1 Hz. Transient absorption spectroscopy (TAS) measurements were performed on a Helios (Ultrafast systems) spectrometers using a regeneratively amplified femtosecond Ti:sapphire laser system (Spitfire Pro-F1KXP, Spectra-Physics; frequency, 1 kHz; max pulse energy, ~8 mJ; pulse width, 120 fs) at room temperature. For the TAS sample preparation of the pristine hematite, ten pieces of Fe_2_O_3_ nanoflakes with the same experimental condition were scratched from the iron foils into a 5 ml sample storage using a plastic dropper. Then, 3 ml deionized water was added into the storage, and was ultrasonic treated for 30 min to maintain high dispersion of the sample. For comparison, we conducted the TAS measurement of the deionized water to eliminate its disturbance during the measurement. The TAS sample preparations of NPs Pt/Fe_2_O_3_ and SAs Pt:Fe_2_O_3_ were used the same treatment of the pristine Fe_2_O_3_. The data were analyzed through commercial software (Surface Xplorer, Ultrafast Systems). An individual three-exponential decay model was used to calculate the fits of the decay. The amplitude weighted average lifetime (*τ*_av_) can be fitted using the following equation of *τ*_av=_$\frac{{{{{{\rm{SUM}}}}}}[{Ai}\times \tau i{]}}{{{{{{\rm{SUM}}}}}}[{Ai}{]}}$, where *A*_i_ is the amplitude of the component with lifetime (*τ*_i_), and *τ*_i_ is the amplitude weighted lifetime.

### Simulation methodology

Geometry optimization, electronic structure, adiabatic molecular dynamic (MD), and nonadiabatic (NA) coupling calculations were performed using Vienna ab initio simulation package (VASP) software^53^. The electron exchange-correlation and electron-ion core interactions were treated with the generalized gradient approximation of Perdew–Burke–Ernzerhof (PBE)^54^ and projector-augmented-wave (PAW)^55^ approaches, respectively. Typically, the Fe 3d strong correlated electrons cannot be well-described by the standard DFT method. Therefore, to correct the strongly correlated electronic nature of Fe 3d-electrons, we applied the on-site Coulomb correction (U = 5 eV for Fe 3d orbitals) to accurately describe the band gap of Fe_2_O_3_ (~2.0 eV), which agrees well with the data (2.1 eV) calculated using the same functional method^56^. The plane wave cutoff energy was set to 500 eV. The weak van der Waals interactions were described with the Grimme DFT-D3 methdsh^57^. The geometry optimizations were carried out at Γ-point because a large supercell was used. The electronic structure calculations were performed on 2 × 2 × 1 grid for k-point sampling. After the geometry optimization, all systems were heated to 300 K by repeated velocity scaling. Then, 4 ps microcanonical ensemble adiabatic MD trajectories were obtained. The nonadiabatic molecular dynamic (NAMD) simulations were calculated with the decoherence-induced surface hopping (DISH) method^58^ implemented within the time-dependent Kohn–Sham density functional framework^59–61^, which were performed using the PYthon eXtension for Ab Initio Dynamics (PYXAID) code^62,63^.

The four-electron OER occurs based on Equations (1)–(4), where the * represents the surfaces:

1 $$ \ast+{{{{{{\rm{H}}}}}}}_{2}{{{{{\rm{O}}}}}}\to*{{{{{\rm{OH}}}}}}+{{{{{{\rm{e}}}}}}}^{-}+{{{{{{\rm{H}}}}}}}^{+}$$

2 $$*{{{{{\rm{OH}}}}}}\to*{{{{{\rm{O}}}}}}+{{{{{{\rm{e}}}}}}}^{-}+{{{{{{\rm{H}}}}}}}^{+}$$

3 $${{{{{{\rm{H}}}}}}}_{2}{{{{{\rm{O}}}}}}+\ast {{{{{\rm{O}}}}}}\to \,*{{{{{\rm{OOH}}}}}}+{{{{{{\rm{e}}}}}}}^{-}+{{{{{{\rm{H}}}}}}}^{+}$$

4 $$*{{{{{\rm{OOH}}}}}}\to \ast+{{{{{{\rm{O}}}}}}}_{2}+{{{{{{\rm{e}}}}}}}^{-}+{{{{{{\rm{H}}}}}}}^{+}$$

The Gibbs free energy $G$ was calculated with the followed equation of $G=\,E+\,{E}_{{ZPE}}-{TS}-{eU}$. $E$, ${E}_{{ZPE}}$, $S,$ $U,$ and ${T}$ correspond single point energy, zero-point energy, entropy, potential versus standard hydrogen electrode, and temperature (298.15 K), respectively. The overpotential (*η*) toward OER was computed using the equation (*U* = 0 V) of *η* $=\frac{{{\max }}\left\{\Delta G1,\Delta G2,\Delta G3,\Delta G4\right\}}{e}-1.23.$ The $\Delta G1,\Delta G2,\Delta G3,\,{{{{{\rm{and}}}}}}\,\Delta G4$ represent the Gibbs free energy difference for elementary reactions.

## Supplementary information

Supporting Information

## Data Availability

The data that support the findings of this study are available from the corresponding authors upon reasonable request. Source data are provided with this paper.

## Source data

Source Data
