# Supplementary material for: Single-atomic-site platinum steers photogenerated charge carrier lifetime of hematite nanoflakes for photoelectrochemical water splitting
Source: Nat Commun. 2023 May 8;14:2640. doi: 10.1038/s41467-023-38343-6 (PMC10167323; doi:10.1038/s41467-023-38343-6)
Supplement: Supplementary file 1 — Supporting Information [file 41467_2023_38343_MOESM1_ESM.pdf]

Supporting Information for

Single-atomic-site platinum steers photogenerated charge carrier lifetime of hematite nanoflakes for photoelectrochemical water splitting

Rui-Ting Gao,[1] Jiangwei Zhang,[1] Tomohiko Nakajima,[2] Jinlu He,[3]* Xianhu Liu,[4] Xueyuan Zhang,[5] Lei Wang,[1,]* & Limin Wu [1,6]*

E-mail: wanglei@imu.edu.cn (L.W.); hejinlu@imu.edu.cn (J.H.); wlm@imu.edu.cn (L.M.W.)

Affiliations: [1] College of Chemistry and Chemical Engineering, College of Energy Material and Chemistry, Inner Mongolia University, Hohhot 010021, China. [2] Advanced Manufacturing Research Institute, National Institute of Advanced Industrial Science and Technology, Tsukuba Central 5, 1-1-1 Higashi, Tsukuba, Ibaraki 305-8565, Japan. [3] College of Chemistry and Chemical Engineering, Inner Mongolia University, Hohhot 010021, China. [4] Key Laboratory of Materials Processing and Mold, Ministry of Education, Zhengzhou University, Zhengzhou 450002, China. [5] State Key Laboratory of Chemo/Biosensing and Chemometrics, College of Chemistry and Chemical Engineering, Hunan University, Changsha 410082, China. [6] Department of Materials Science and State Key Laboratory of Molecular Engineering of Polymers, Fudan University, Shanghai 200433, China.

Contents:

1. ***Supplementary Figures 1-47***
2. ***Supplementary Tables 1-9***

Supplementary Figures and Discussion

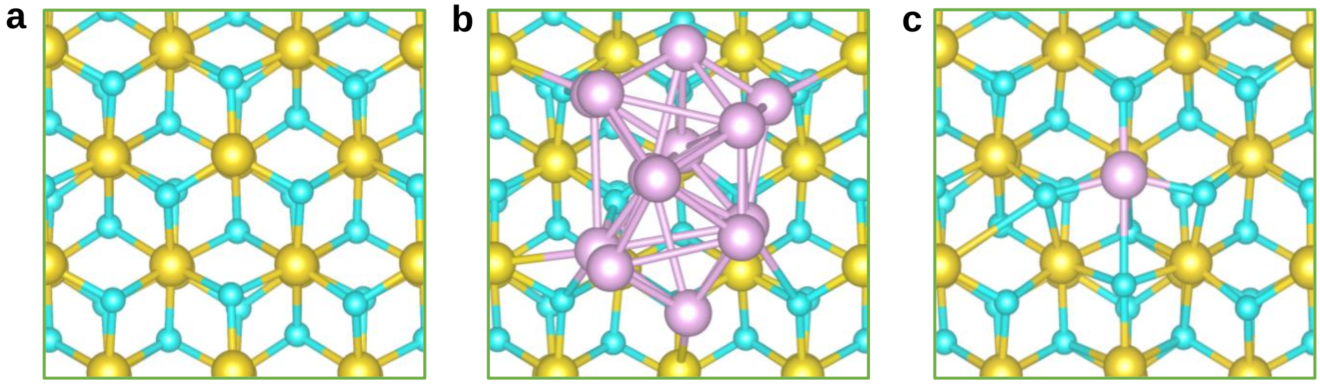

Supplementary Fig. 1 a-c Structural modes of **a** Fe_2O_3, **b** NPs Pt/Fe_2O_3, and **c** SAs Pt:Fe_2O_3.

Discussion: The periodic hexagonal supercell of Fe_2O_3 system (Supplementary Fig. 1a) was constructed with 3 × 3 expansion of using the unit cell Fe_2O_3 with space group of *R -3 c:H*, which contains 81 oxygen and 54 iron atoms. The optimized lattice parameters of Fe_2O_3 surface are a = b = 15.02 Å, *c* = 21.24 Å. The thickness of Fe_2O_3 surface contains four layers of iron atoms. The NPs Pt/Fe_2O_3 (Supplementary Fig. 1b) was created by supported Pt_{13} nanocluster supported on Fe_2O_3 surface, and Pt_{13} nanocluster has a typical spherical shape and ~5.5 Å diameter. We choose the common Pt_{13} nanocluster because it has been extensively studied in many studies.[1-3] We placed the Pt_{13} nanocluster on the middle of Fe_2O_3 surface to prevent the nanocluster from collapsing when it on the boundary of Fe_2O_3 structure. The SAs Pt:Fe_2O_3 (Supplementary Fig. 1c) systems was built by replacing a surface iron atom with a platinum atom and there is only one substitution because the surface Fe atomic sites are equivalent. In addition, the SAs Pt doping is stable than loading due to the formed strong Pt-O bonds. The distance between different SAs Pt atoms is 15.02 Å. Thus, the lateral interactions can be well prevented.

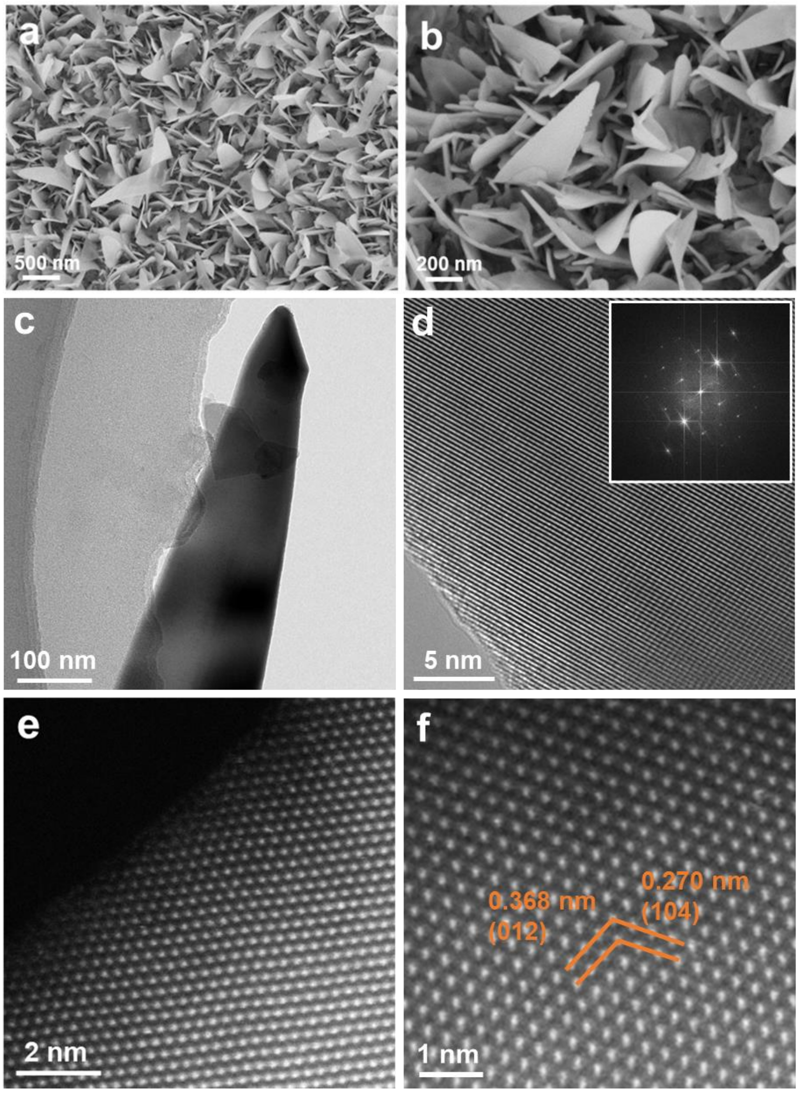

Supplementary Fig. 2 a, b SEM, **c, d** TEM, and **e, f** high resolution HAADF-STEM images of Fe_2O_3. Inset of **d** shows the FFT pattern.

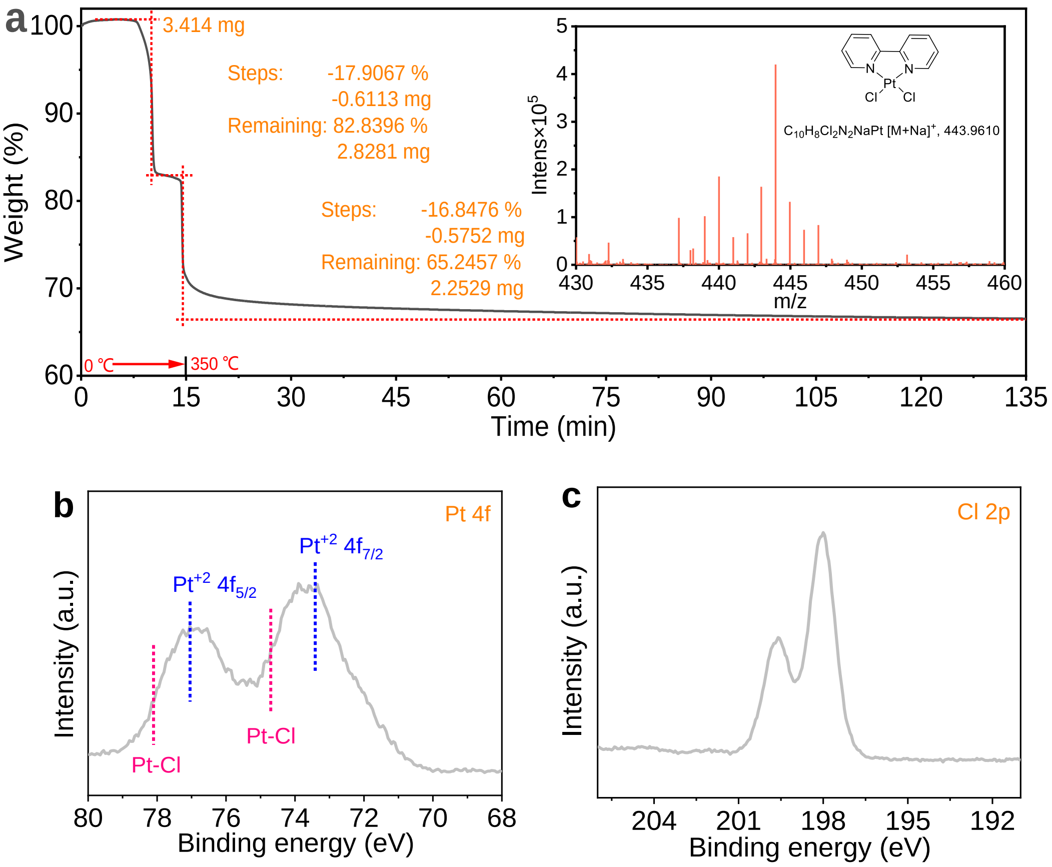

Supplementary Fig. 3 **a** Thermogravimetry of organic complexes containing Pt (inset: Mass spectrometry); **b, c** XPS spectra of **b** Pt *4f* and **c** Cl *2p* of hexachloroplatinic acid hexahydrate and 2,2-bipyridine.

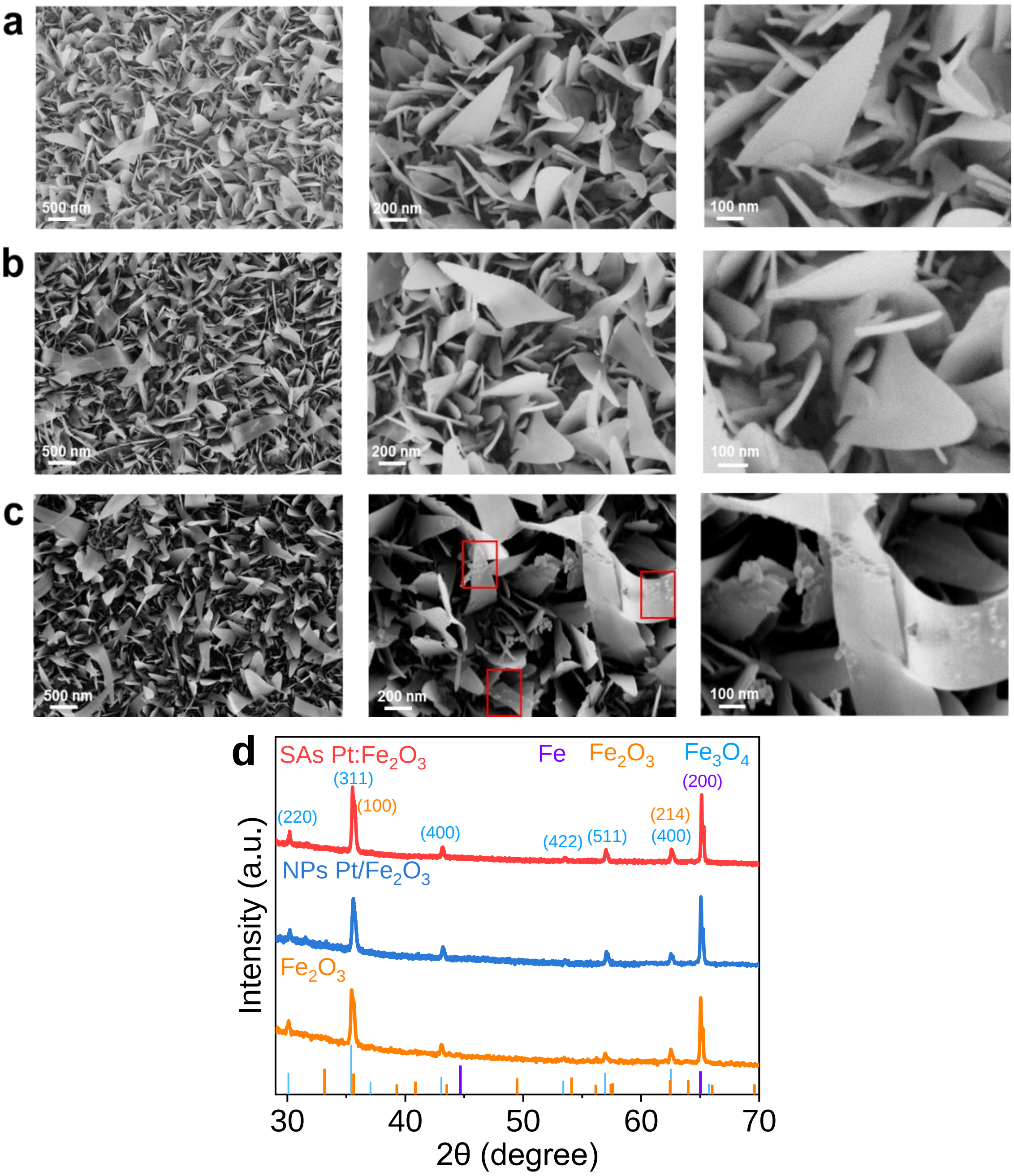

Supplementary Fig. 4 a-c SEM images of **a** pristine Fe_2O_3, **b** SAs Pt:Fe_2O_3, and **c** NPs Pt/Fe_2O_3; **d** XRD patterns of corresponding samples.

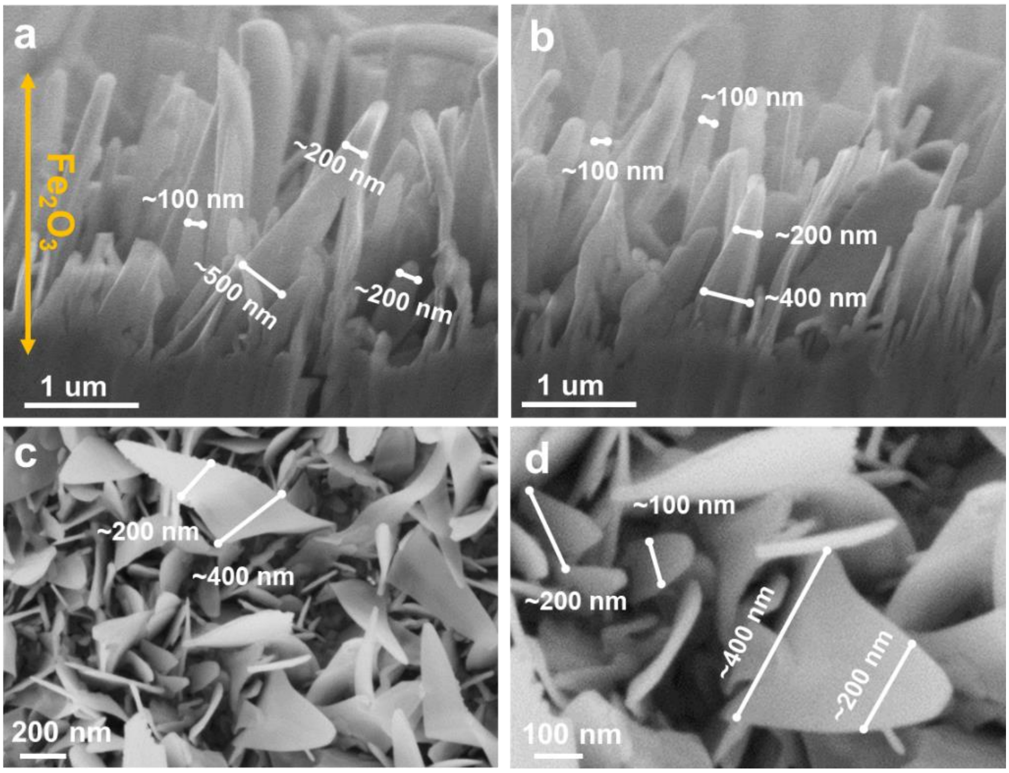

Supplementary Fig. 5 a, b Cross-sectional and **c, d** top-view SEM images of SAs Pt:Fe_2O_3.

Discussion: Because the growth orientations of the nanoflakes is different during thermal annealing, cross-sectional SEM observations present the different directions. Even though one sample was chosen in different area, the flakes have the different orientations and sizes, in which some flakes grow to a long length of ~2.5 μm, and some grow to a short length of ~500 nm.

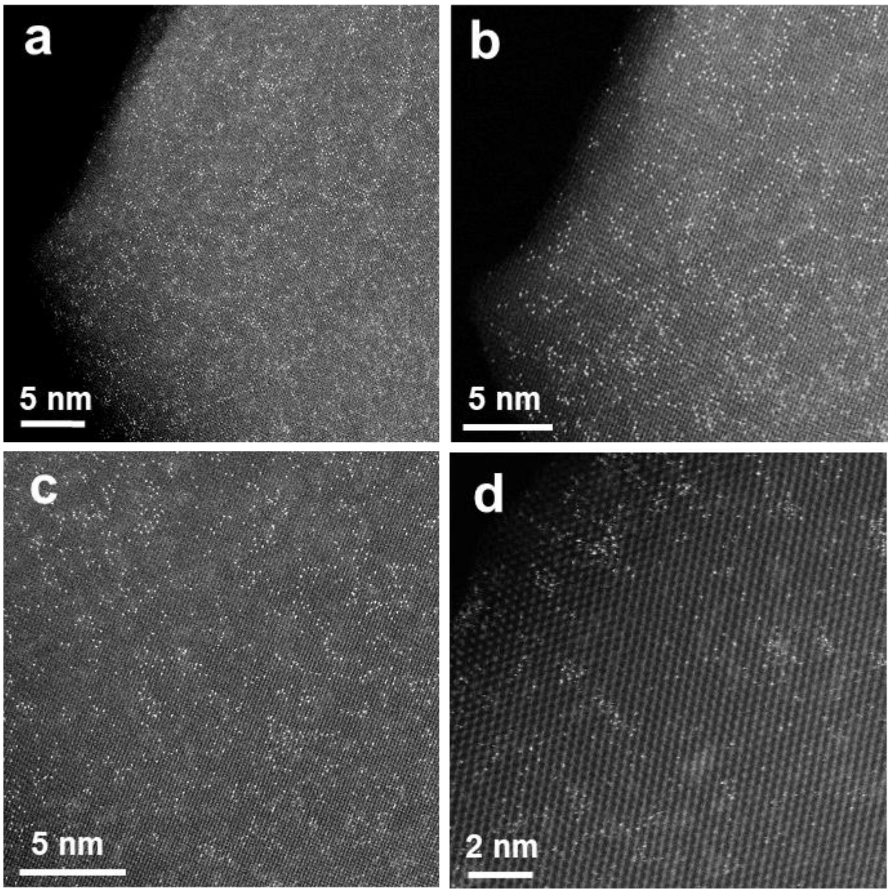

Supplementary Fig. 6 a-d HAADF-STEM images of SAs Pt:Fe_2O_3.

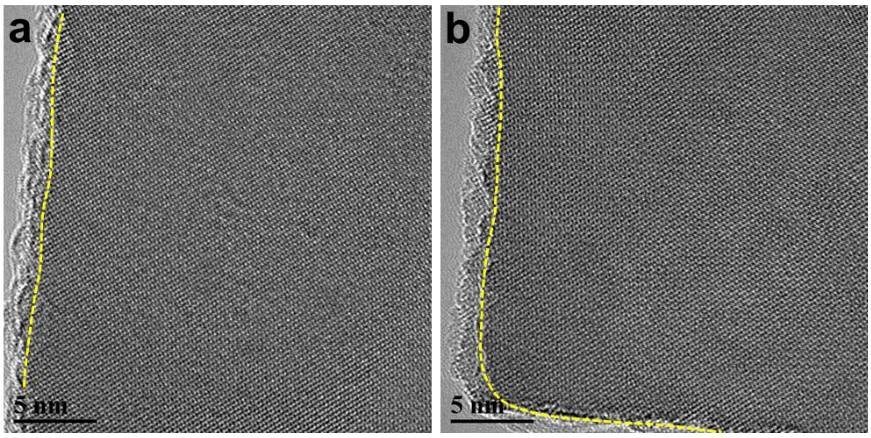

Supplementary Fig. 7 a, b HRTEM images of SAs Pt:Fe_2O_3.

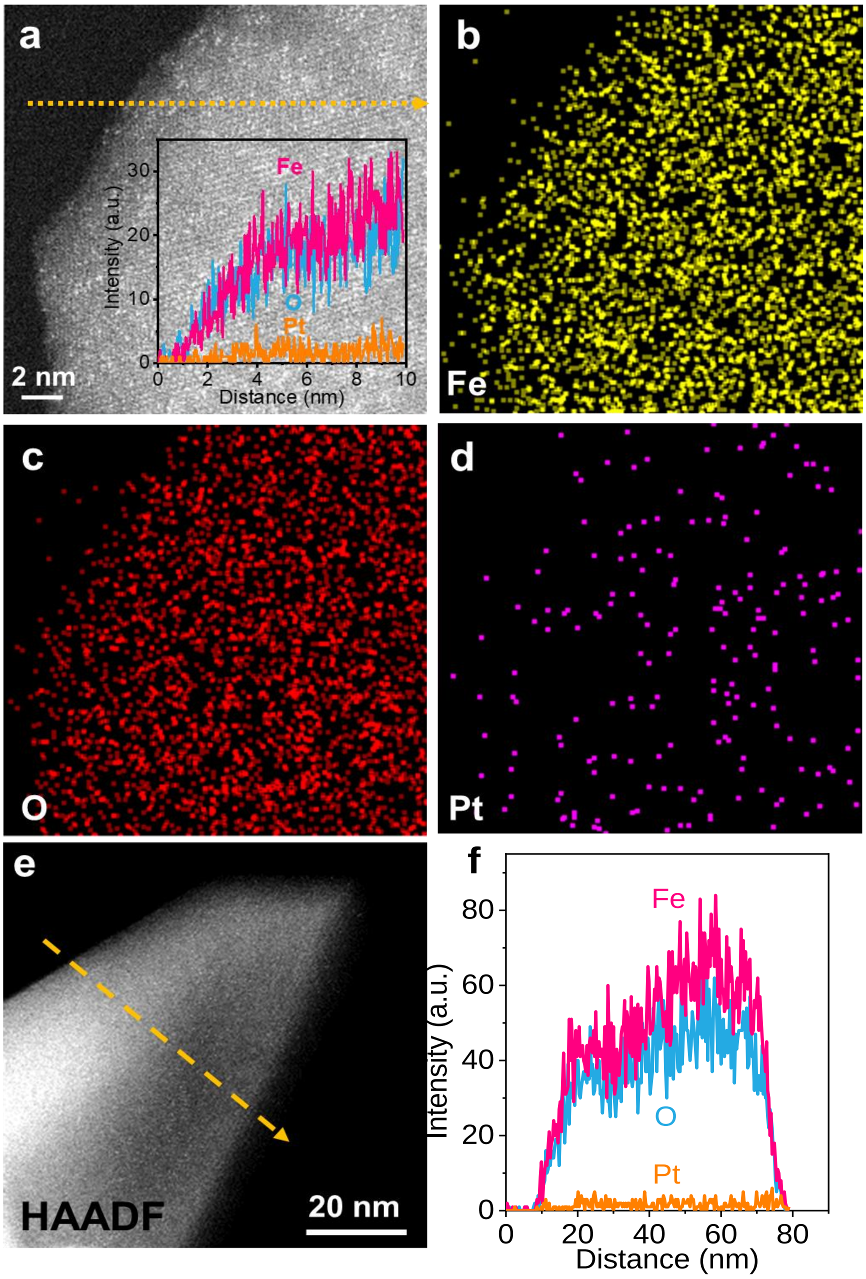

Supplementary Fig. 8 a-d Elemental mapping of SAs Pt:Fe_2O_3. Inset of **a** shows EELS linear scanning along the dashed arrow; **e, f** EELS linear scanning along the dashed arrow in **e**.

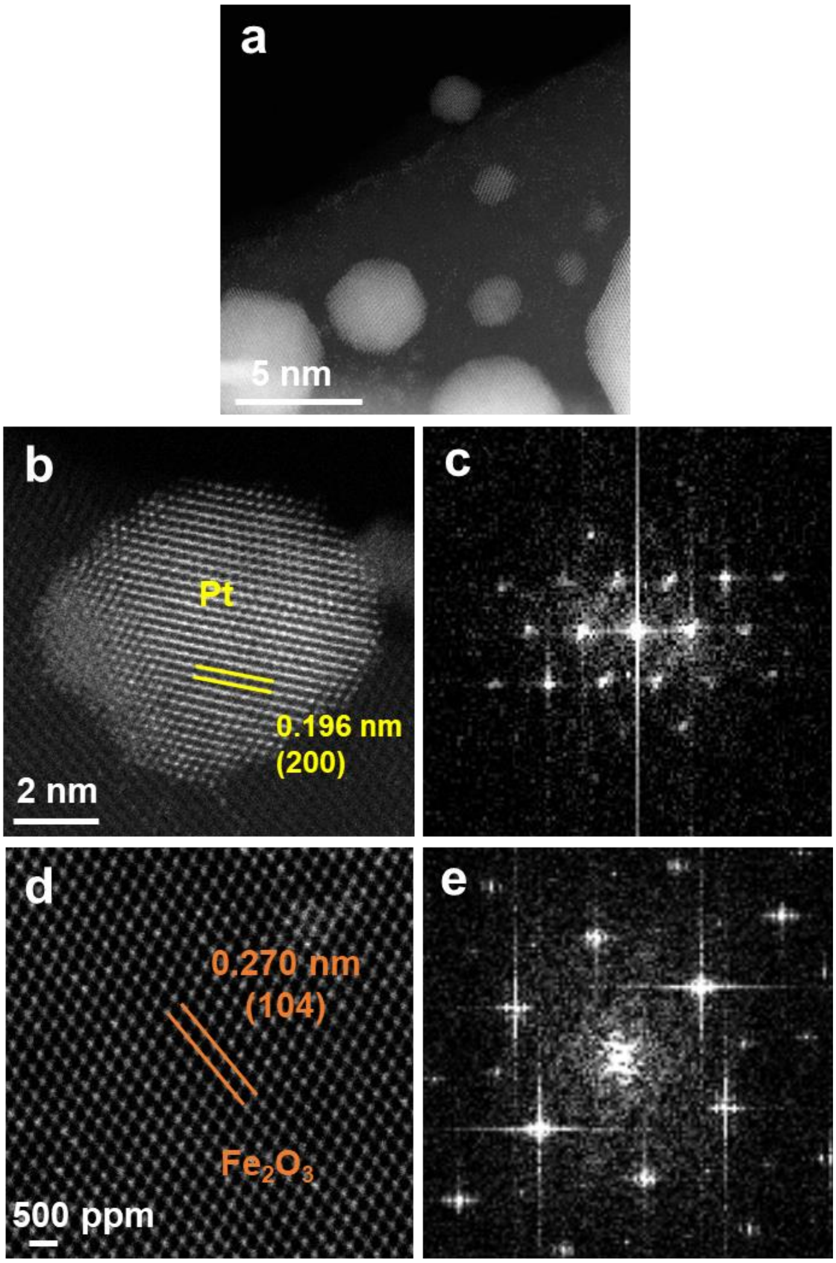

Supplementary Fig. 9 a, b, d HAADF-STEM images and **c, e** FFT patterns of NPs Pt/Fe_2O_3.

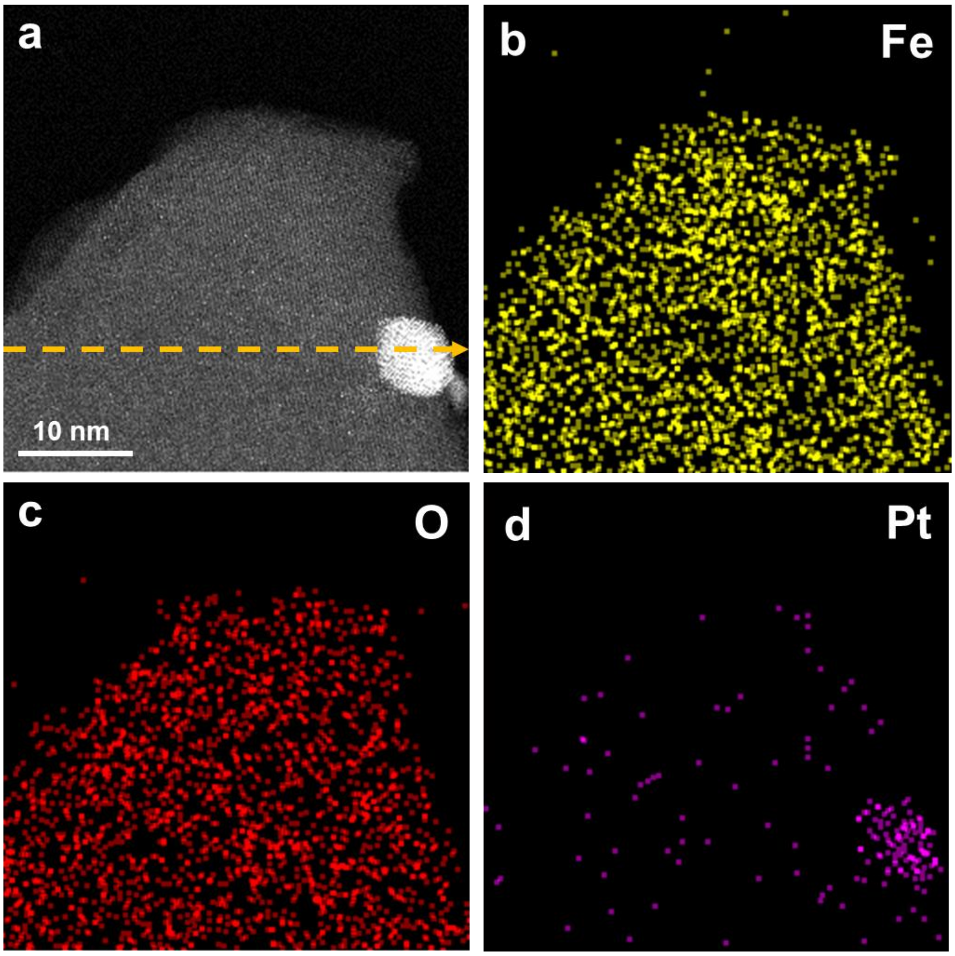

Supplementary Fig. 10 a-d Elemental mapping of NPs Pt/Fe_2O_3.

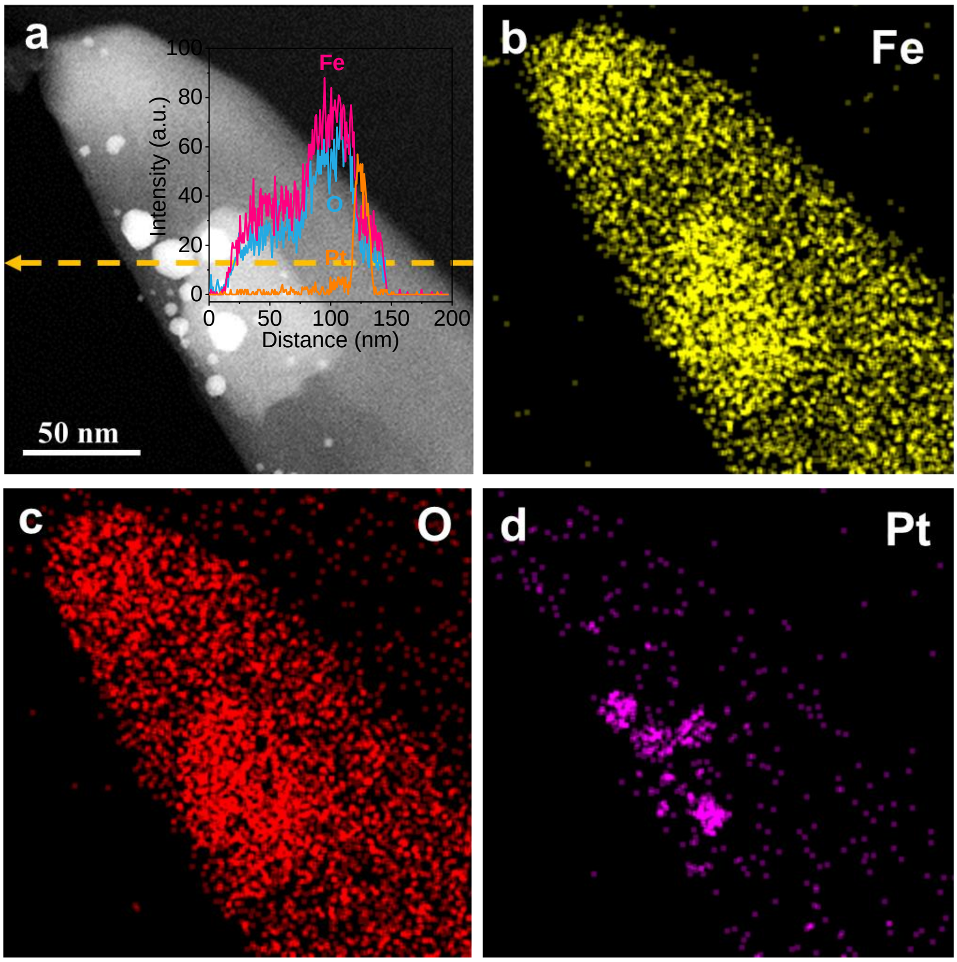

Supplementary Fig. 11 a-d Elemental mapping for NPs Pt/Fe_2O_3. Inset of **a** shows EELS linear scanning along the dashed arrow.

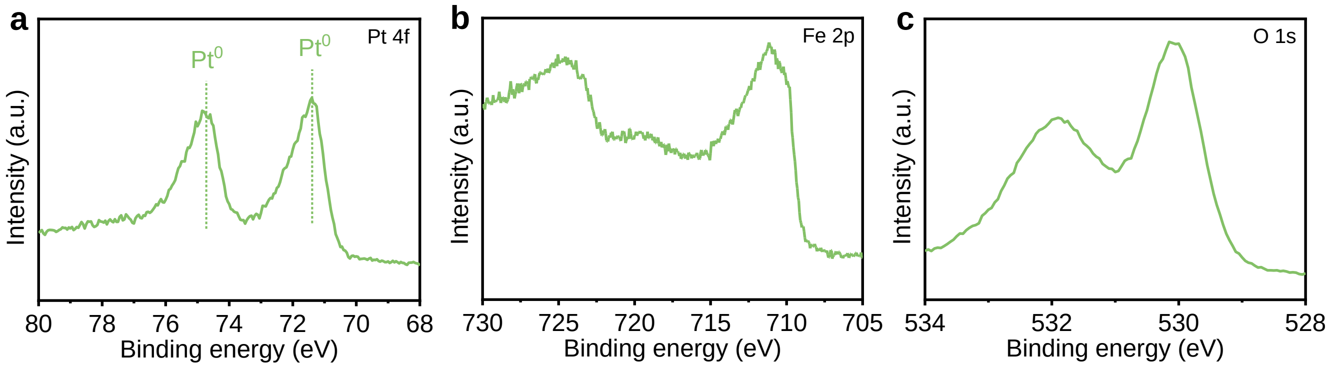

Supplementary Fig. 12 a Pt *4f*, **b** Fe *2p*, and **c** O *1s* XPS spectra for Pt:Fe_2O_3. Fe_2O_3 nanoflakes were immersed in the prescursor containing hexachloroplatinic acid hexahydrate without 2,2-bipyridine as the ligand. The sample was subsequesntly annealed under Ar atmosphere at 330 °C to obtain Pt doped Fe_2O_3.

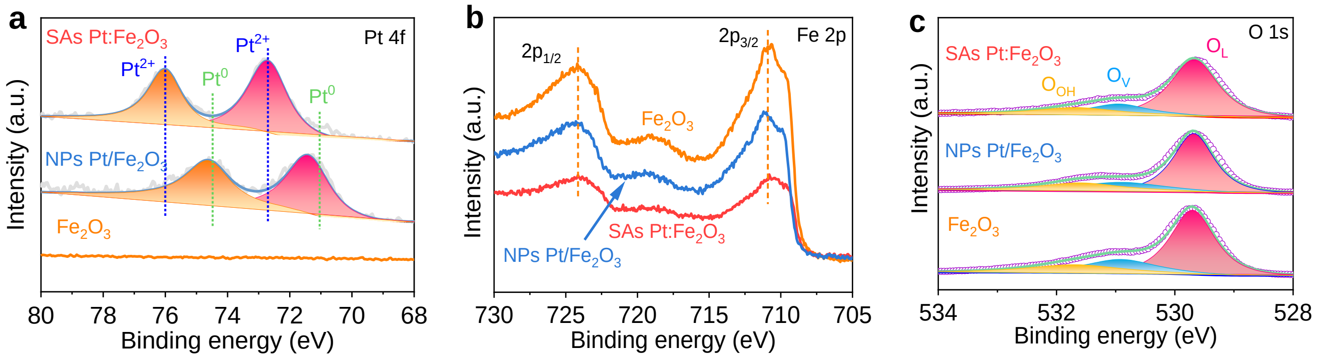

d

Sample	Fe (at.%)	O (at.%)	Pt (at.%)
Fe_2O_3	29.99	70.01	0
NPs Pt/Fe_2O_3	26.71	64.02	9.27
SAs Pt:Fe_2O_3	22.86	67.17	9.96

Supplementary Fig. 13 a Pt *4f*, **b** Fe *2p*, and **c** O *1s* XPS spectra for Fe_2O_3, NPs Pt/Fe_2O_3, and SAs Pt:Fe_2O_3; **d** atomic element ratios of corresponding samples from XPS analysis.

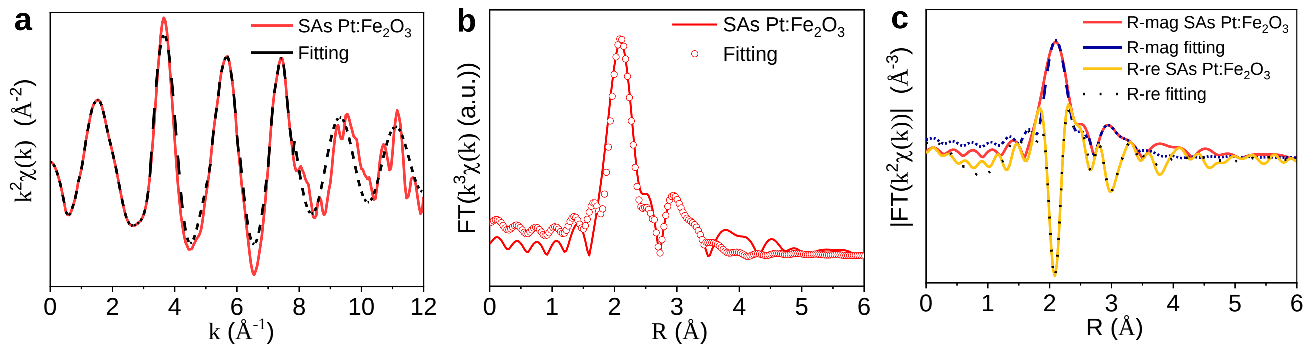

Supplementary Fig. 14 a-c Pt *K*-edge EXAFS data and fit for SAs Pt:Fe_2O_3 in **a** *k*-space and **b, c** *R*-space.

XAFS measurements and analysis. Pt *K*-edge analysis was performed with Si (111) crystal monochromators at the BL14W1 beamlines at the Shanghai Synchrotron Radiation Facility (SSRF) (Shanghai, China). Before the analysis at the beamline, samples were pressed into thin sheets with 1 cm in diameter and sealed using Kapton tape film. The XAFS spectra were recorded at room temperature using a 4-channel Silicon Drift Detector (SDD) Bruker 5040. Pt *K*-edge extended X-ray absorption fine structure (EXAFS) spectra were recorded in fluorescence mode. Negligible changes in the line-shape and peak position of Pt *K*-edge XANES spectra were observed between two scans taken for a specific sample. The acquired EXAFS data were processed according to the standard procedures using the ATHENA module of Demeter software packages. The EXAFS spectra were obtained by subtracting the post-edge background from the overall absorption and then normalizing with respect to the edge-jump step. Subsequently, the $\chi(k)$ data of were Fourier transformed to real (R) space using a hanning windows (dk=1.0 $Å^{-1}$) to separate the EXAFS contributions from different coordination shells. To obtain the quantitative structural parameters around central atoms, least-squares curve parameter fitting was performed using the ARTEMIS module of Demeter software packages.

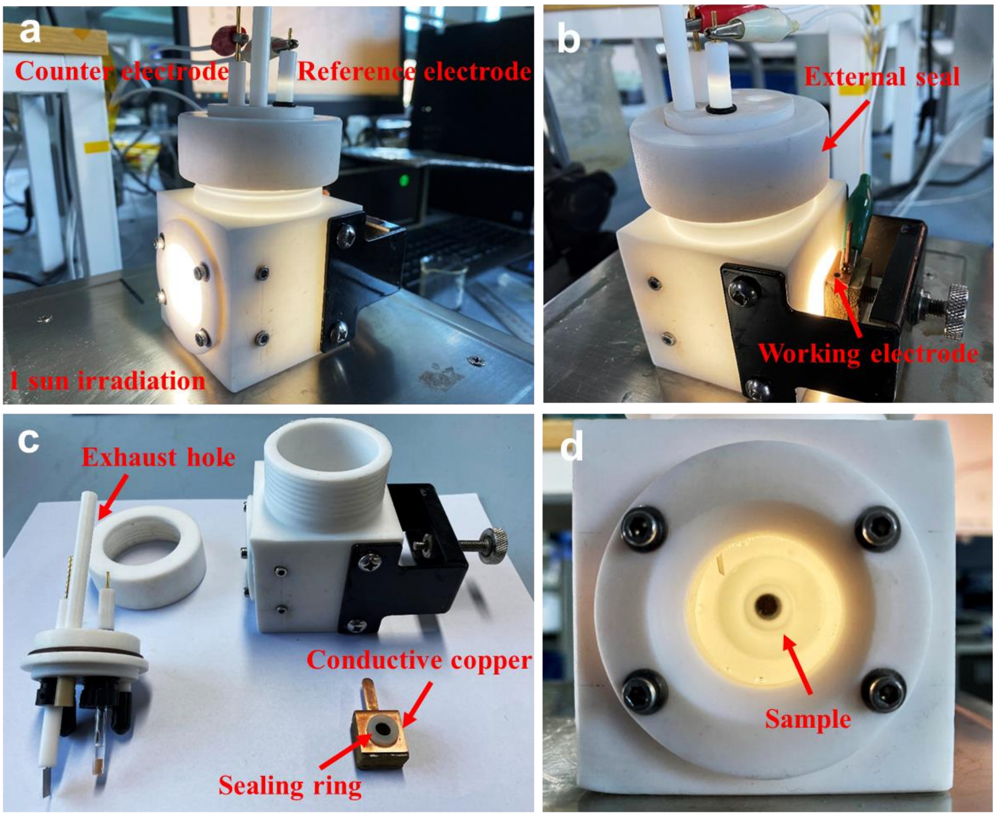

Supplementary Fig. 15 a-d A typical PEC system in this work.

Discussion: For PEC measurement, the sample surfaces of both sides were covered with the hematite nanoflakes after thermal annealing treatment. The back side of the prepared sample was polished until the metal Fe substrate was exposed. We then placed the sample on a conductive Cu foil, where the polished side was touched to the Cu foil. And, a sealing O'ring was put on the sample with the tested side. The prepared photoelectrode was assembled on a PEC cell and pressed down to ensure that the device was sealed completely. Next, we would check the tested sample whether it put in the center of the light window, and meanwhile removing the gases bubbles around the sample for the following measurement.

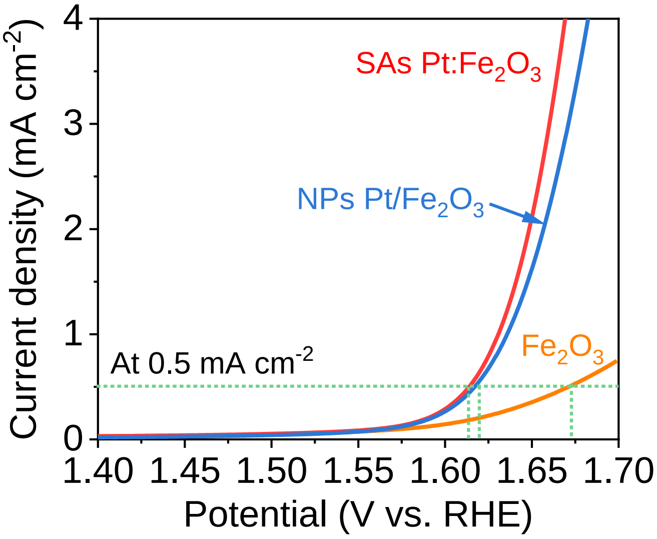

Supplementary Fig. 16 *J-V* curves of Fe_2O_3, NPs Pt/Fe_2O_3, and SAs Pt:Fe_2O_3 under dark.

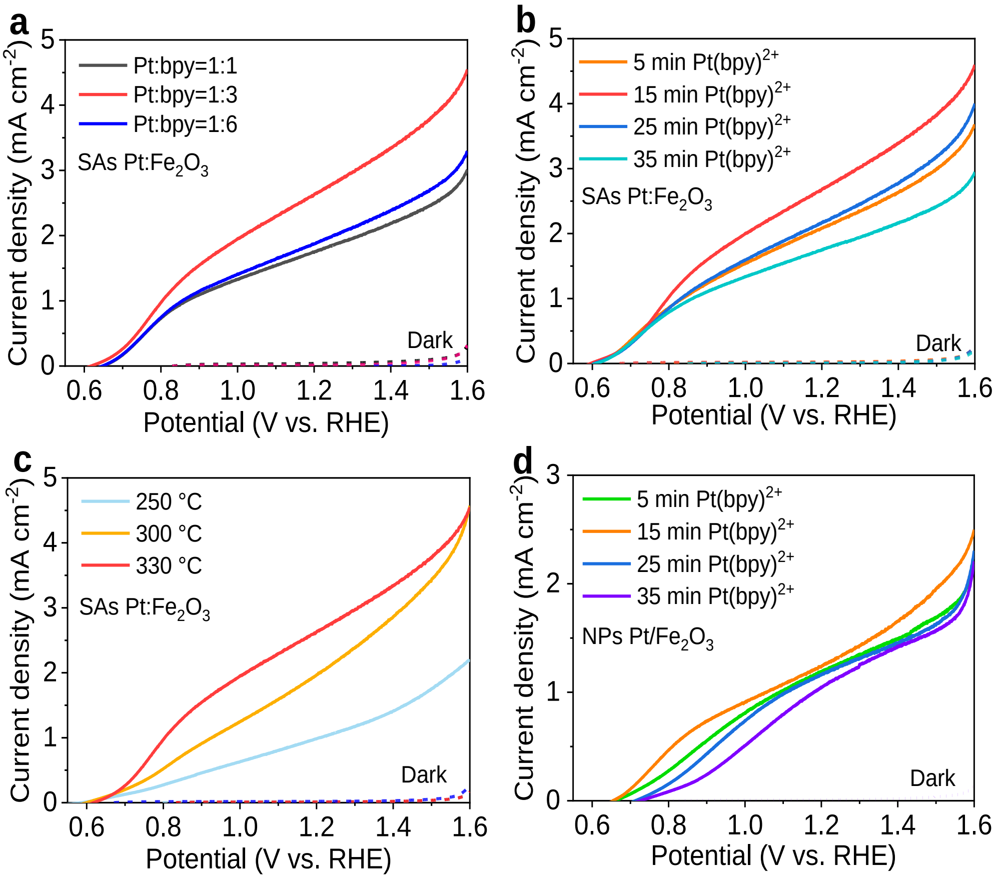

Supplementary Fig. 17 a *J-V* curves of SAs Pt:Fe_2O_3 with various molar ratios (1:1, 1:3, and 1:6) of hexachloroplatinic acid hexahydrate and 2,2-bipyridine; **b** *J-V* curves of SAs Pt:Fe_2O_3 immersion in Pt(bpy)$^{2+}$ solution for different times (5 min, 15 min, 25 min, and 35 min); **c** *J-V* curves for SAs Pt:Fe_2O_3 annealing at various annealing temperatures (250 °C, 300 °C, and 330 °C); **d** *J-V* curves of NPs Pt/Fe_2O_3 immersion for different times (5 min, 15 min, 25 min, and 35 min) annealing at 400 °C. All measurements were performed in 1 M KOH under AM 1.5G illumination (100 mW cm^{-2}).

Discussion: As the annealing temperature is further increased to 400 °C, NPs Pt were doped into the hematite as shown in Supplementary Fig. 9. NPs Pt/Fe_2O_3 was optimized with various loading of Pt (Supplementary Fig. 17d). It can be assured that the optimum NPs Pt/Fe_2O_3 still displays a low PEC activity in contrast to the SAs Pt:Fe_2O_3 (Supplementary Fig. 17b).

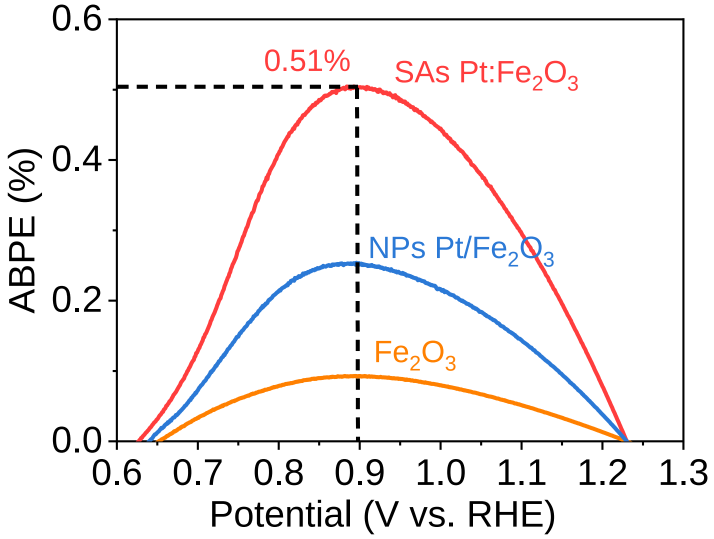

Supplementary Fig. 18 ABPE curves of Fe_2O_3, NPs Pt/Fe_2O_3, and SAs Pt:Fe_2O_3.

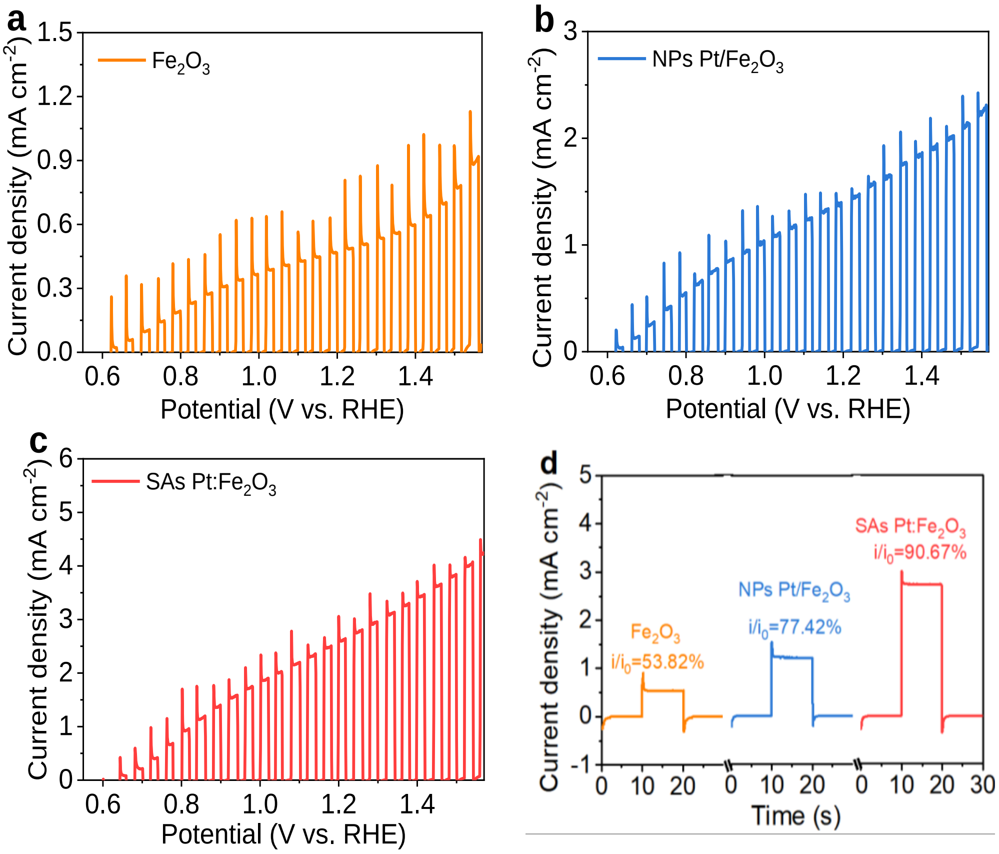

Supplementary Fig. 19 a-c Chopped *J-V* curves of **a** Fe_2O_3, **b** NPs Pt/Fe_2O_3, and **c** SAs Pt:Fe_2O_3; **d** transient photocurrents of corresponding samples at 1.23 V_{RHE}. All measurements were performed in 1 M KOH under AM 1.5G illumination (100 mW cm^{-2}).

Discussion: Transient photoresponse implies the capable extraction of photoholes onto the surface. As seen from Supplementary Fig. 19d, the transient photocurrent for SAs Pt:Fe_2O_3 (i/i_0=91%) is significantly higher than those of the NPs Pt/Fe_2O_3 (i/i_0=77%) and the pristine Fe_2O_3 (i/i_0=54%), suggesting that atomic doping facilitates the reduction of charge recombination and efficiently transfers the charge carrier into the water oxidation reaction.

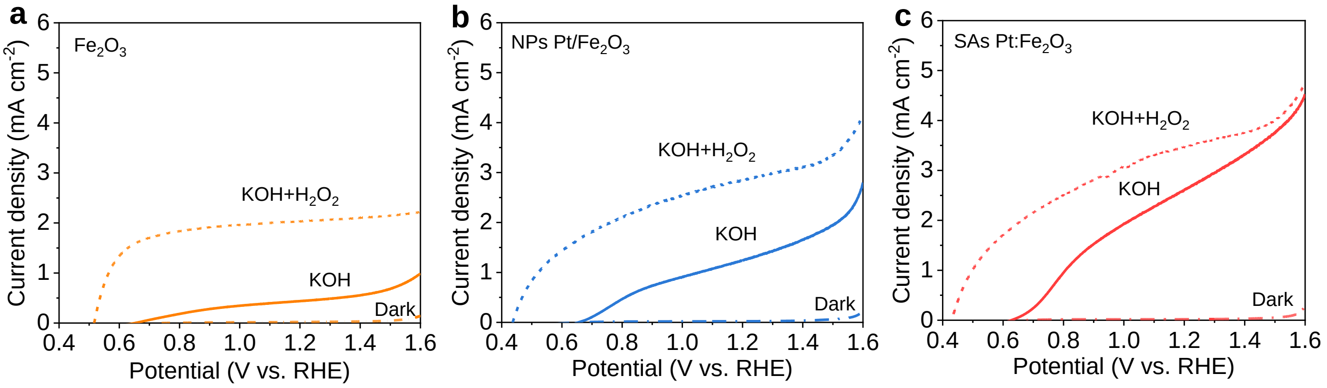

Supplementary Fig. 20 a-c *J-V* curves of **a** Fe_2O_3, **b** NPs Pt/Fe_2O_3, and **c** SAs Pt:Fe_2O_3 in 1 M KOH without and with the addition of H_2O_2 under AM 1.5G illumination (100 mW cm^{-2}).

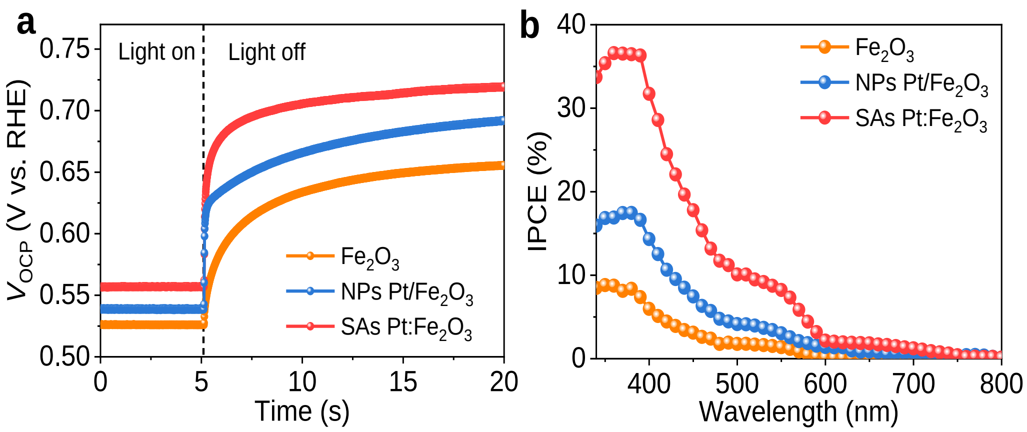

Supplementary Fig. 21 a OCP transient decay curves and **b** IPCE values of Fe_2O_3, NPs Pt/Fe_2O_3, and SAs Pt:Fe_2O_3.

Discussion: To compare the charge recombination rate at the photoanode/electrolyte junction, the charge carrier transfer lifetime (τ) can be calculated by the equation of $\tau_n = -\frac{k_B T}{e}\left(\frac{d\mathrm{OCP}}{dt}\right)^{-1}$ based on the previous reports.[4,5] The τ_n, k_B, T, e, and dOCP/dt are the carrier transfer lifetime, Boltzmann's constant, temperature, charge of single electron, and derivative of the OCP transient decay, respectively. OCP curves are obtained by the electrochemical tests without and with illumination. The OCP transient decay assesses the surface recombination between trapped electrons and reaction intermediates, and this process is very fast in the range of ns-ms, while the scale of OCP decay is usually in several minutes. OCP is very positive in dark owing to a largely upward band bending, while OCP will be more cathodic under illumination owing to flattening of the energy band by photoexcited carriers. ΔOCP (OCP_{dark}-OCP_{light}) as photovoltage corresponds to the amount of band bending under illumination in contrast to that in the dark condition. SAs Pt doping on hematite promotes ΔOCP value in regard to that of NPs Pt doping on hematite. At the transient time from illumination (quasi-equilibrium of flattened energy band) to the dark (equilibrium of bent energy band), the charge recombination strongly depends on the spatial charges built in the photoanode/electrolyte junction. The strong band bending enables a large amount of spatial charges in the depletion region and thus significant charge recombination occurs at the transient when the illumination was removed.

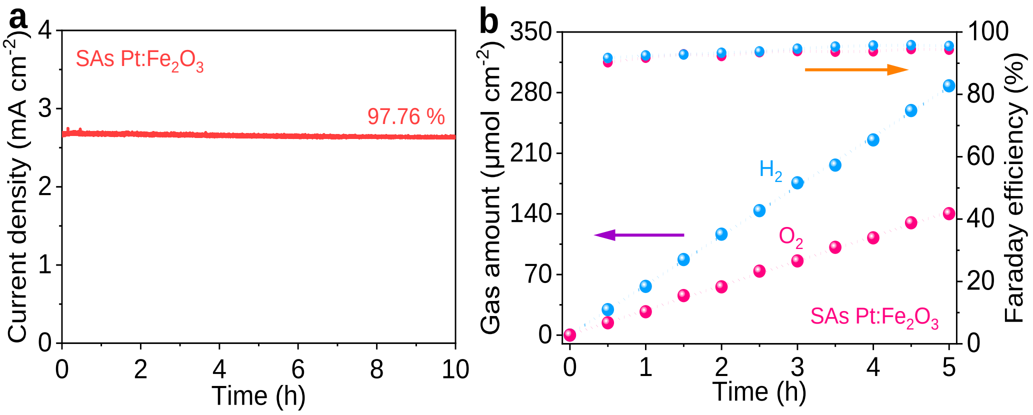

Supplementary Fig. 22 a Stability measurement of SAs Pt:Fe_2O_3 applied at 1.23 V_{RHE} in 1 M KOH under AM 1.5G illumination (100 mW cm^{-2}); **b** gas evolutions detected by gas chromatography and faradaic efficiency of SAs Pt:Fe_2O_3 at 1.23 V_{RHE} for 5 h.

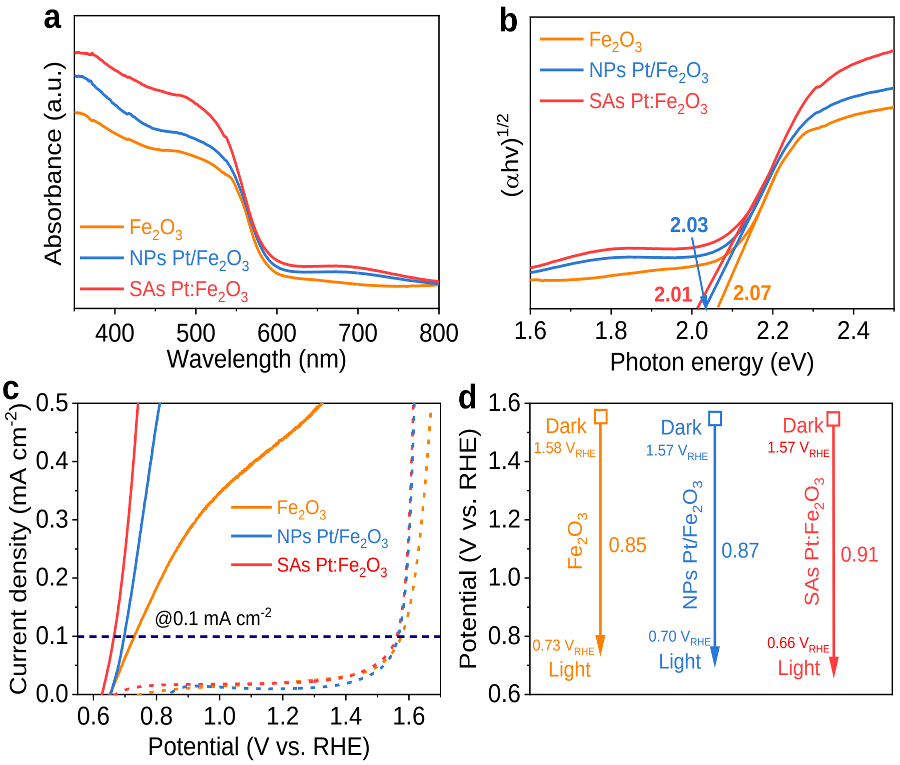

Supplementary Fig. 23 a UV-vis absorption spectra and **b** Tauc plots of UV-Vis adsorption spectra for Fe_2O_3, NPs P/:Fe_2O_3, and SAs Pt:Fe_2O_3; **c** Potential shifts between dark and light current density of Fe_2O_3, NPs Pt/Fe_2O_3, and SAs Pt:Fe_2O_3; **d** extracted photovoltages of Fe_2O_3, NPs Pt/Fe_2O_3, and SAs Pt:Fe_2O_3.

Discussion: We arrange the photovoltage values for all samples at the same current density of 0.1 mA cm^{-2} (Supplementary Fig. 23c), and the *Δ*potential value increases from 0.85 (pristine Fe_2O_3) to 0.87 (NPs Pt/Fe_2O_3) and 0.91 (SAs Pt:Fe_2O_3), consistent with the OCP transient decay curves (Supplementary Fig. 21a). ultraviolet-visible (UV-vis) absorption and ultraviolet photoelectron spectroscopy (UPS) are used to accurately determine the band edge position of NPs Pt/Fe_2O_3 and SAs Pt:Fe_2O_3. Specifically, the band gaps of the samples were determined by UV-vis spectrum. The Tauc diagram of UV-vis spectra shows that the optical band gaps of the pristine Fe_2O_3, NPs Pt/Fe_2O_3, and SAs Pt:Fe_2O_3 are 2.07, 2.03, and 2.01 eV, respectively. The decrease of band gap after Pt doping may be due to the increase of oxygen defects in Fe_2O_3 samples.[6] The work functions of the pristine Fe_2O_3, NPs Pt/Fe_2O_3, and SAs Pt:Fe_2O_3 were determined by the following equation:[7,8]

$$W_F = hv - E_{cutoff} \quad (1)$$

in which the excitation energy hv of He I is 21.2 eV, and Ecutoff is the cutoff energy of the secondary electron. The positions of the valence band (EVB) maxima with respect to the Fermi levels were obtained from the onset of valence band photoemission on the low-binding-energy edges (EL) of UPS spectra,[5] as shown in the inset of Fig. R13b.

$$E_F = Ev - W_F \quad (2)$$

$$E_{VB} = E_F - E_L \quad (3)$$

Vacuum level (*E*v= 0 eV), while the difference between the vacuum level and the standard hydrogen electrode potential is 4.5 eV. Together with the UV-vis and UPS data, the detailed band positions for each sample are obtained and summarized in Table R5.

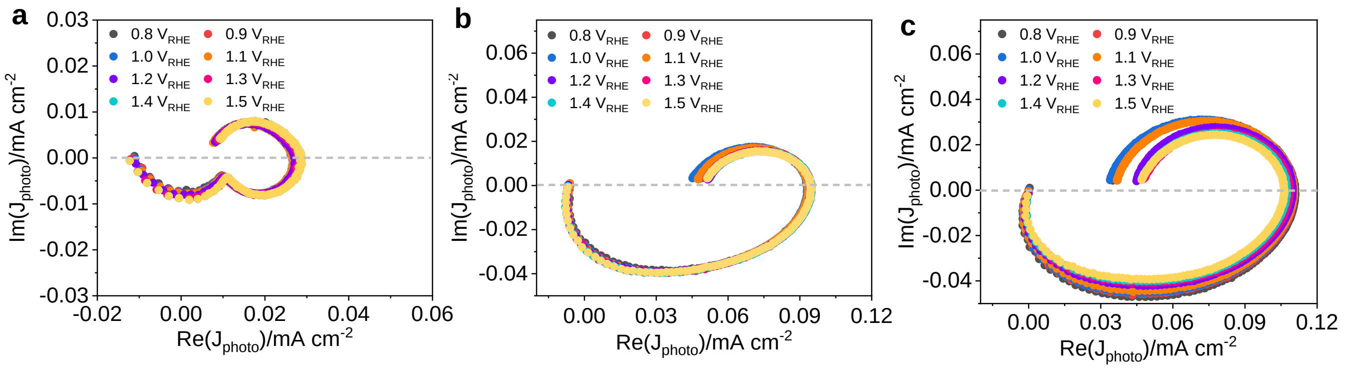

Supplementary Fig. 24 a-c IMPS responses for **a** Fe_2O_3, **b** NPs Pt/Fe_2O_3, and **c** SAs Pt:Fe_2O_3 applied at various voltages (0.8, 0.9, 1.0, 1.1, 1.2, 1.3, 1.4, and 1.5 V_{RHE}) in 1 M KOH.

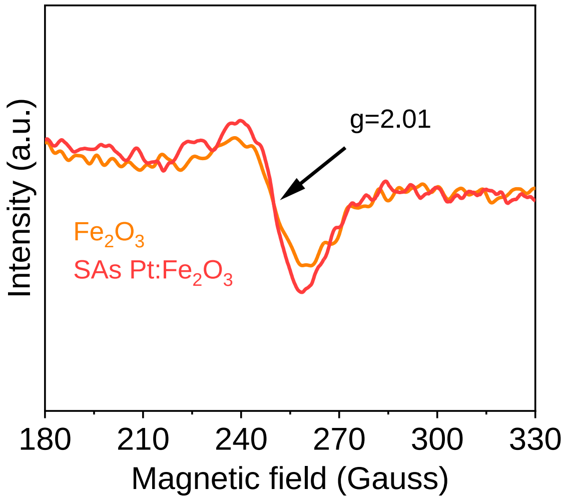

Supplementary Fig. 25 EPR curves of Fe_2O_3 and SAs Pt:Fe_2O_3.

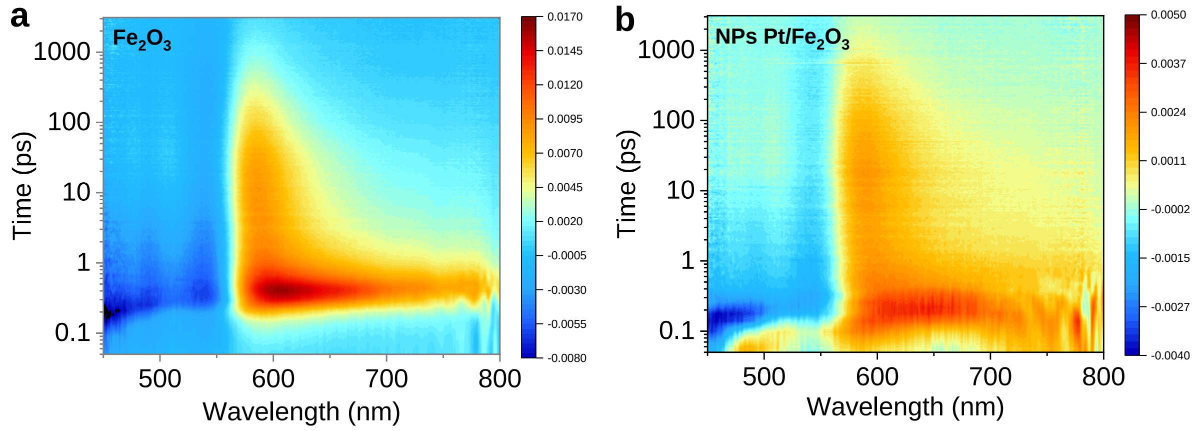

Supplementary Fig. 26 a, b Time-resolved transient absorption spectra of **a** Fe_2O_3 and **b** NPs Pt/Fe_2O_3 when excited with 380 nm light.

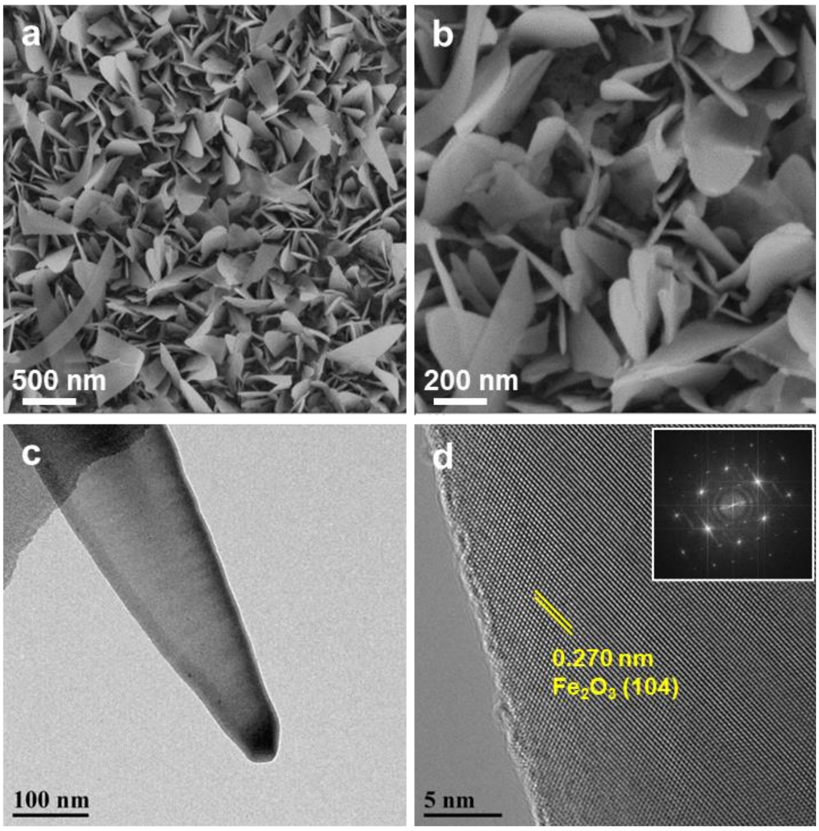

Supplementary Fig. 27 a, b SEM and **c, d** TEM images of SAs Pt:Fe_2O_3-O_V. Inset of **d** shows the FFT pattern.

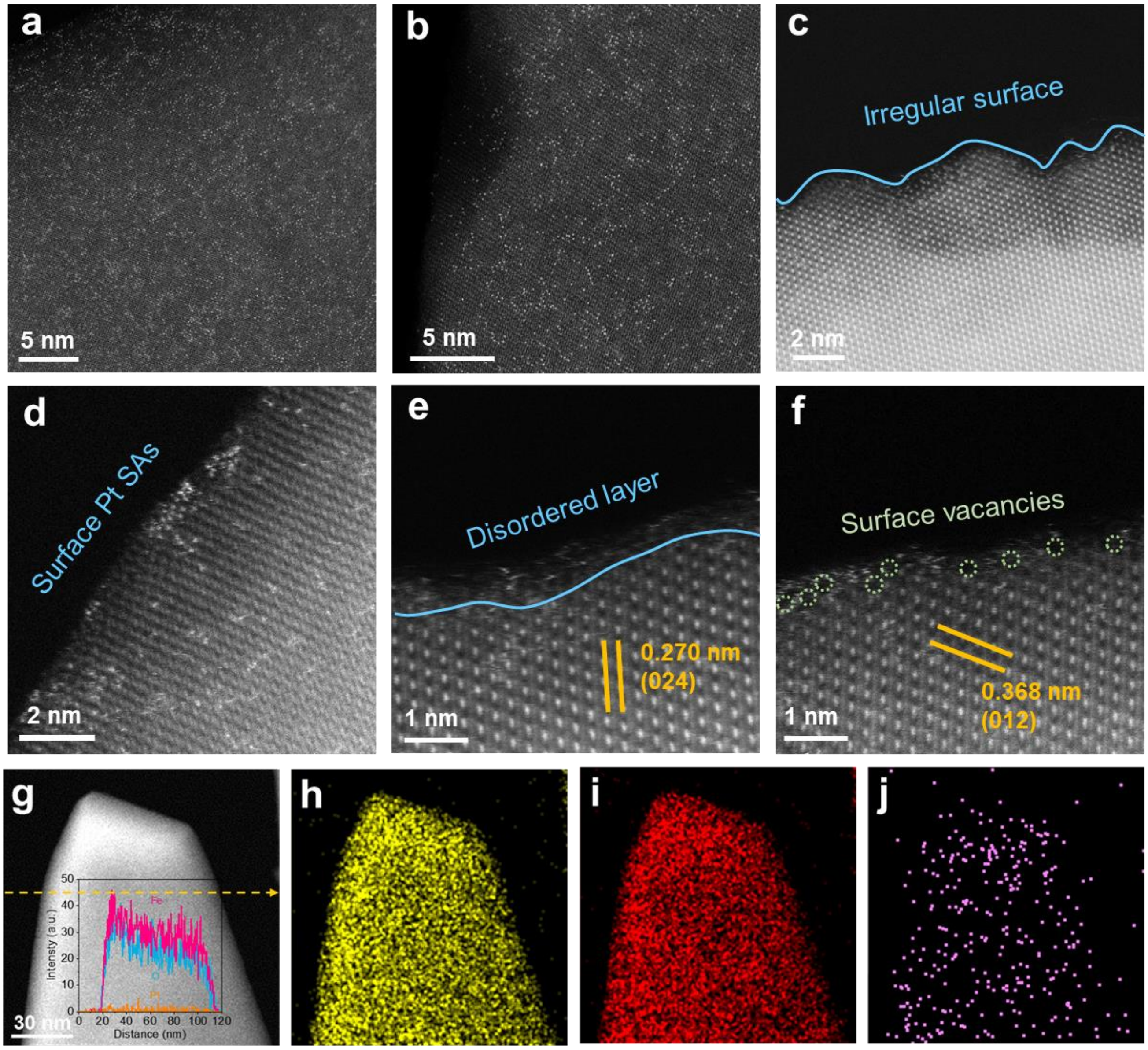

Supplementary Fig. 28 a-f HAADF-STEM images and **g-j** elemental mapping of SAs Pt:Fe_2O_3-O_V. Inset of **g** shows corresponding EELS linear scanning along the dashed arrow.

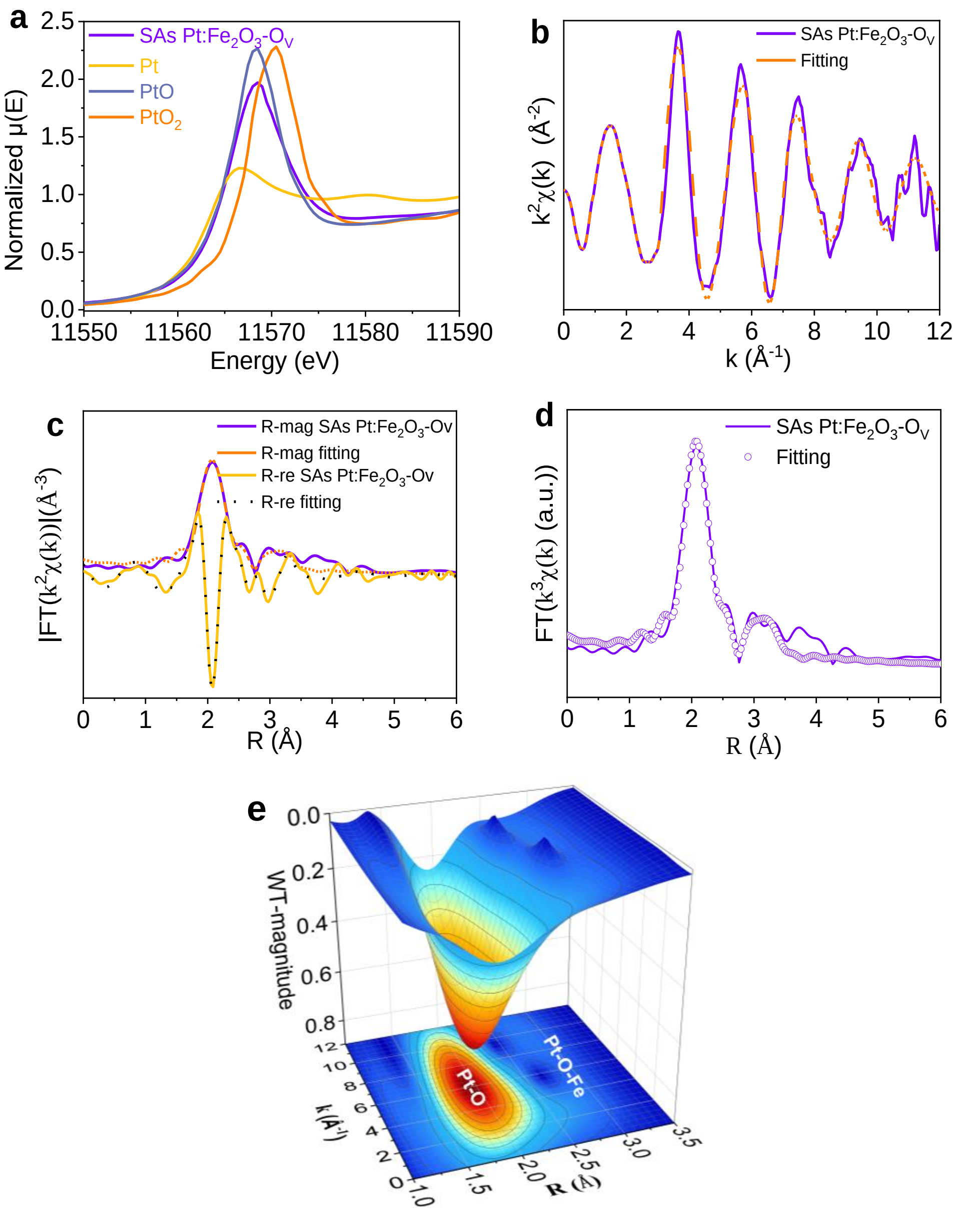

Supplementary Fig. 29 a Pt *K*-edge XANES spectra of SAs Pt:Fe_2O_3-O_V, Pt foil, PtO, and PtO_2; **b-d** Pt *K*-edge EXAFS data and fit for SAs Pt:Fe_2O_3-O_V in **b** *k*-space and **c, d** *R*-space; **e** WT-EXAFS plot of SAs Pt:Fe_2O_3-O_V.

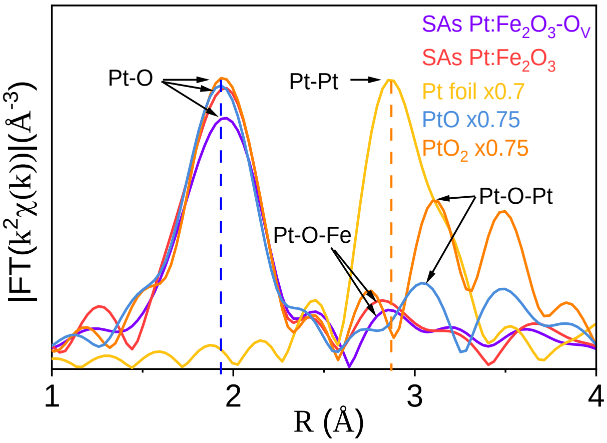

Supplementary Fig. 30 Comparison of Fourier-transformed *R*-space of the experimental Pt *K*-edge EXAFS signals of SAs Pt:Fe_2O_3 and SAs Pt:Fe_2O_3-O_V. No Pt-Cl bond located at 2.24-2.26 Å can be seen, meaning the break of Pt-Cl bond during the annealing.

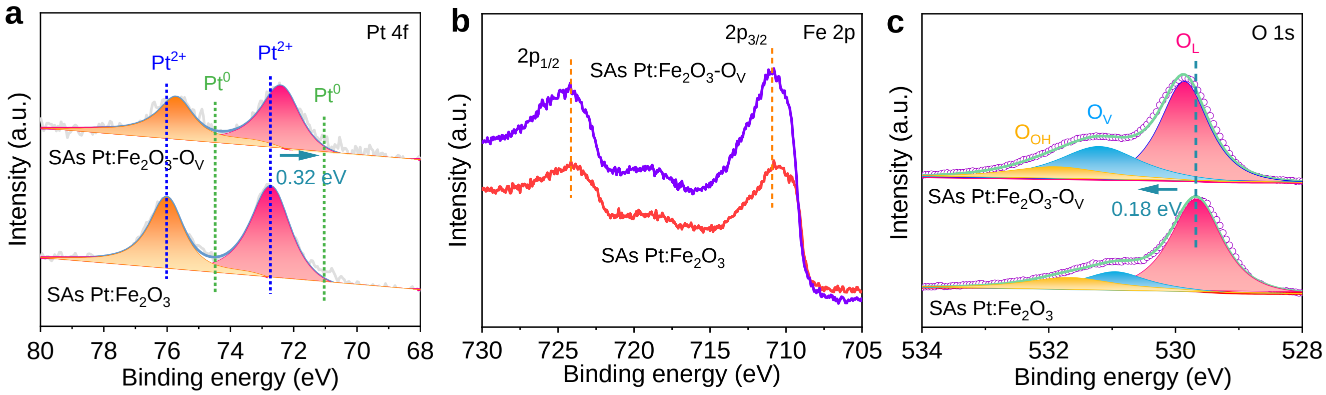

Supplementary Fig. 31 **a** Pt *4f*, **b** Fe *2p*, and **c** O *1s* XPS spectra of SAs Pt:Fe_2O_3 and SAs Pt:Fe_2O_3-O_V.

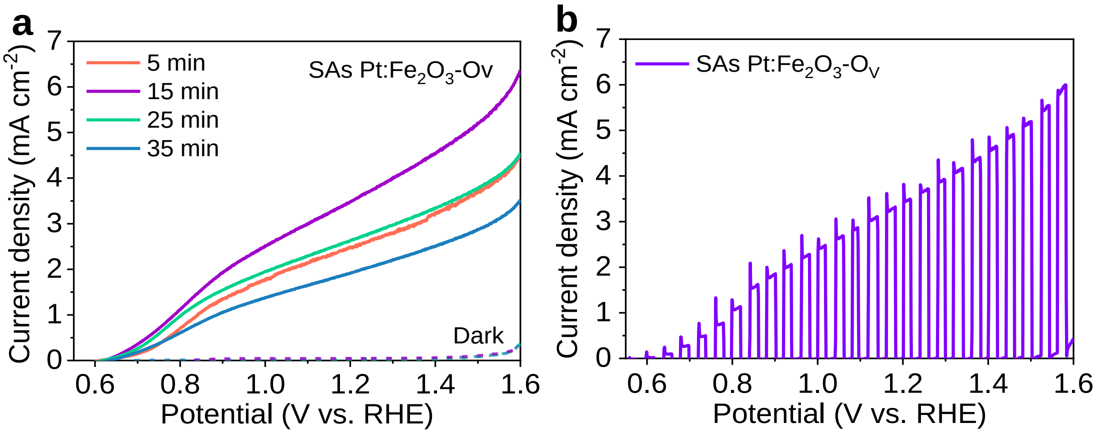

Supplementary Fig. 32 **a** *J-V* curves of SAs Pt:Fe_2O_3-O_V after plasma etching treatment at 20 W for various times (5 min, 15 min, 25 min, and 35 min); **b** chopped *J-V* curve of SAs Pt:Fe_2O_3-O_V. All measurements were performed in 1 M KOH under AM 1.5G illumination (100 mW cm^{-2}).

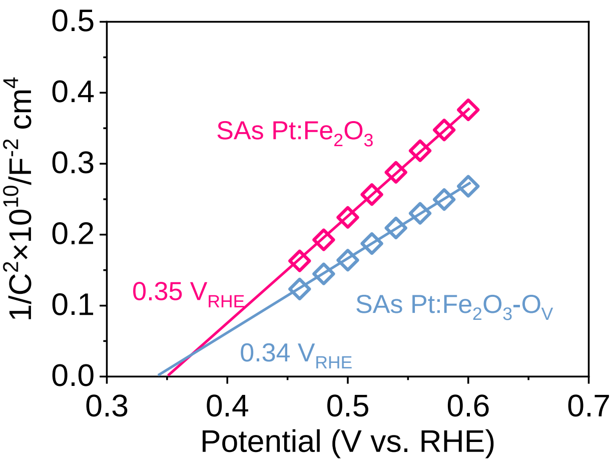

Supplementary Fig. 33 Mott-Schottky curves of SAs Pt:Fe_2O_3 and SAs Pt:Fe_2O_3-O_V.

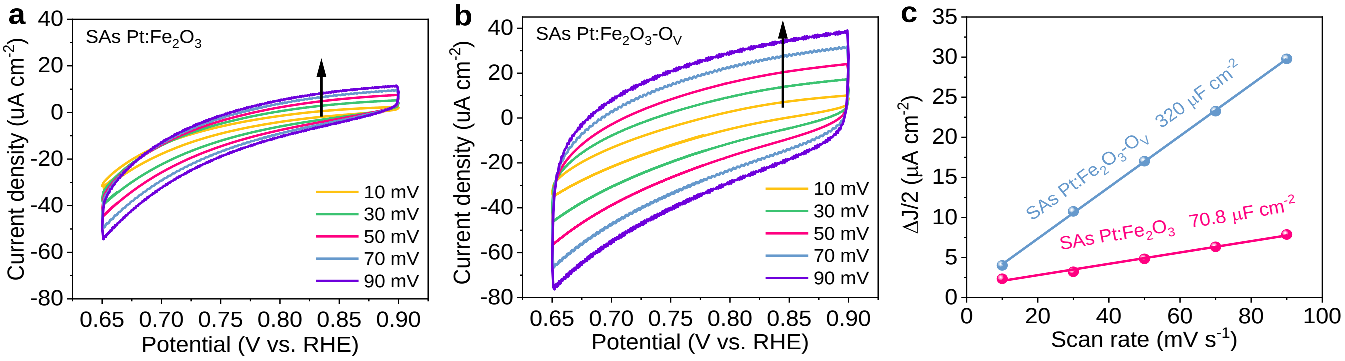

Supplementary Fig. 34 a, b Cyclic voltammetry curves of **a** SAs Pt:Fe_2O_3 and **b** SAs Pt:Fe_2O_3-O_V; **c** relative electrochemical surface areas for the linear relationship between the capacitive current and scan rate for SAs Pt:Fe_2O_3 and SAs Pt:Fe_2O_3-O_V.

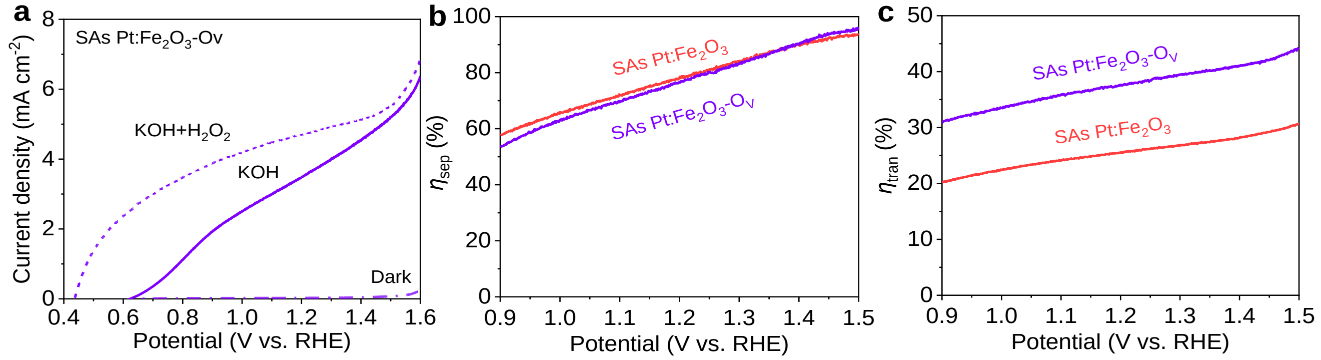

Supplementary Fig. 35 a *J-V* curves of SAs Pt:Fe_2O_3-O_V in 1 M KOH without and with the addition of H_2O_2 under AM 1.5G illumination (100 mW cm^{-2}); **b** charge separation efficiencies and **c** charge transfer efficiencies of SAs Pt:Fe_2O_3 and SAs Pt: Fe_2O_3-O_V.

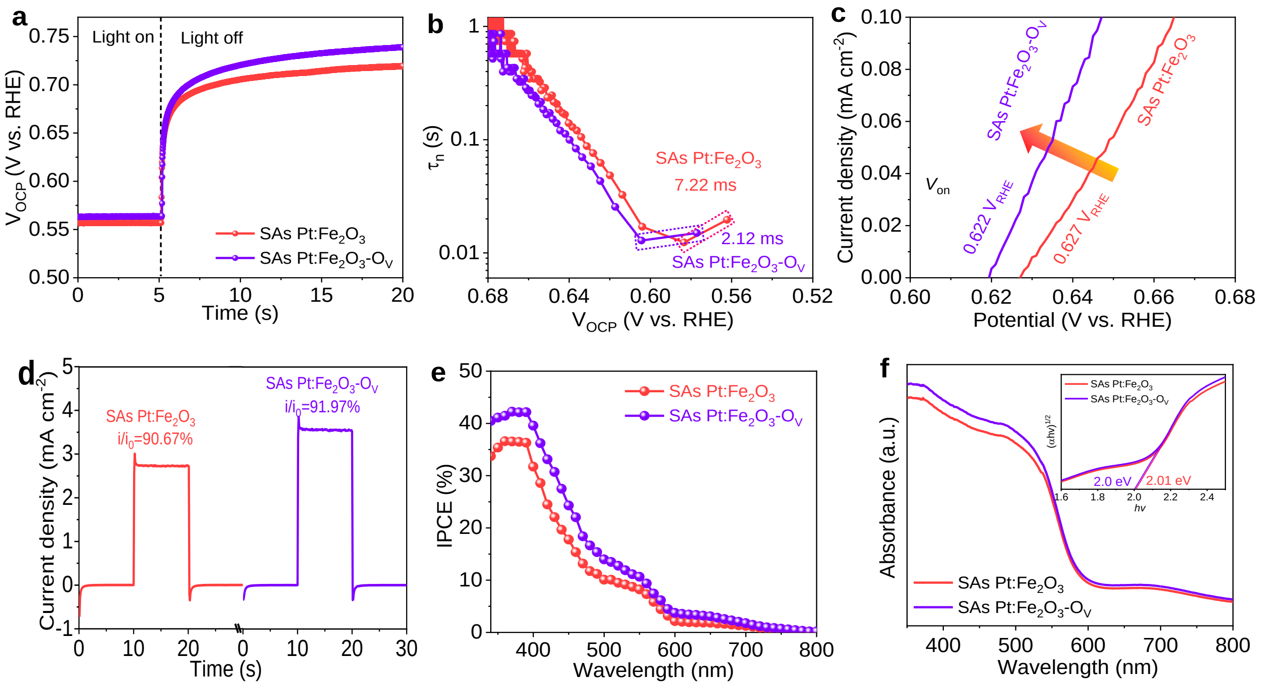

Supplementary Fig. 36 Comparison of electrochemical characterization between SAs Pt:Fe_2O_3 and SAs Pt:Fe_2O_3-O_V: **a** OCP transient decay profiles; **b** OCP-derived carrier transfer lifetimes; **c** extracted V_{on}; **d** transient photocurrent; **e** IPCE values; **f** UV-vis light absorption curves. Inset of **f** shows Tauc plots of UV-vis adsorption spectra.

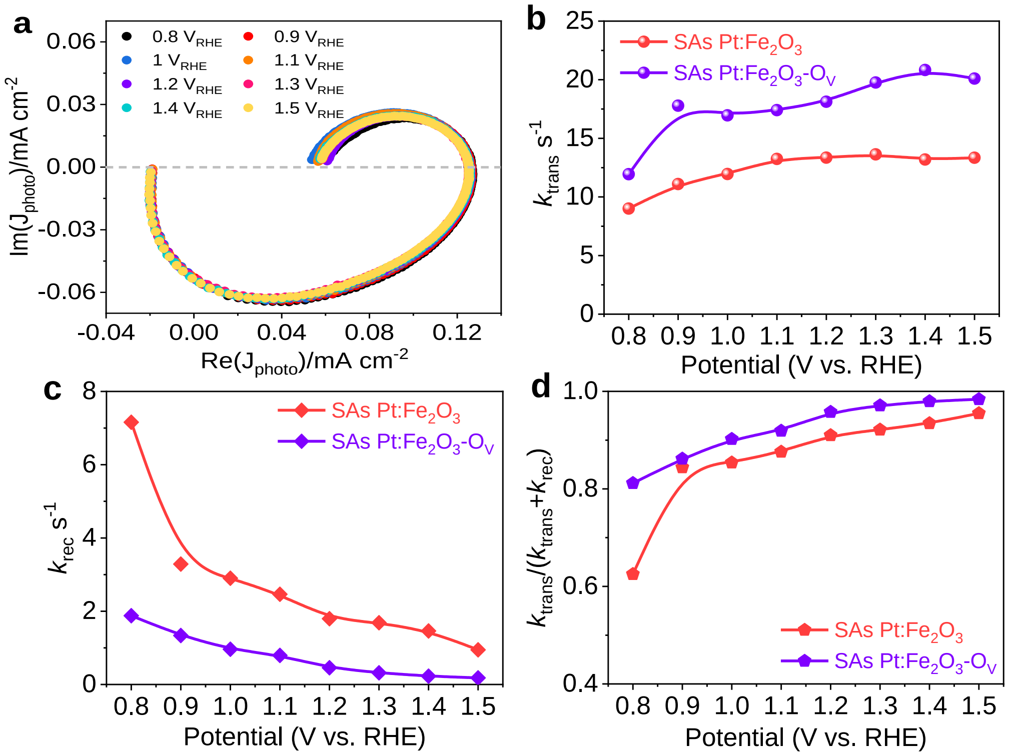

Supplementary Fig. 37 a IMPS responses of SAs Pt:Fe_2O_3-O_V applied at various potentials in 1 M KOH; **b** charge transfer rate constants (k_{trans}), **c** charge recombination rate constants (k_{rec}), and **d** charge transfer efficiencies for SAs Pt:Fe_2O_3 and SAs Pt:Fe_2O_3-O_V extracted from IMPS analysis.

Discussion: Obtained from the IMPS analysis, it can be shown that SAs Pt:Fe_2O_3-O_V has shown the fast charge transfer rate and low charge recombination rate compared to the SAs Pt:Fe_2O_3 phooanode. Meanwhile, the charge transfer efficiency of SAs Pt:Fe_2O_3-O_V is definitely higher than the SAs Pt:Fe_2O_3, confirming the presence of surface oxygen vacancies facilitate the surface charge quickly transfer to the surface for water oxidation reaction.

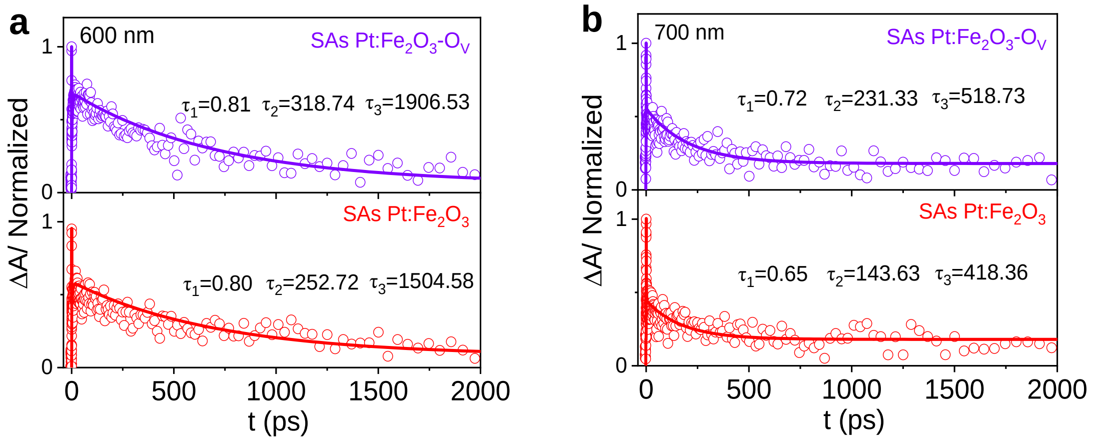

c

Sample	A_1×1000	τ_1 (ps)	A_2×1000	τ_2 (ps)	A_3×1000	τ_3 (ps)	τ_{av} (ps)
SAs Pt:Fe_2O_3-O_V 600 nm	0.49	0.81	0.63	318.74	0.62	1906.53	794.97
SAs Pt:Fe_2O_3-O_V 700 nm	0.28	0.72	0.23	231.33	0.38	518.73	281.49

Supplementary Fig. 38 a, b Transient absorption decays at **a** 600 nm and **b** 700 nm for SAs Pt:Fe_2O_3 and SAs Pt:Fe_2O_3-O_V. SAs Pt:Fe_2O_3 and SAs Pt:Fe_2O_3-O_V excited from 380 nm at a delay of 1 ns. The fits for the decays were calculated with three exponential decay model, shown as the solid lines, whereas the squares represent experimental data; **c** fits for the decays of SAs Pt:Fe_2O_3-O_V.

Discussion: The carrier dynamics for the SAs Pt:Fe_2O_3-O_V photoanode was calculated by fitting the kinetics traces at 600 nm and 700 nm. The charge carrier decay average lifetime of τ_{av} at 600 nm extends from 486.76 ps (SAs Pt:Fe_2O_3) to 794.97 ps for SAs Pt:Fe_2O_3-O_V, and at 700 nm from 155.86 ps (SAs Pt:Fe_2O_3) to 281.49 ps for SAs Pt:Fe_2O_3-O_V.

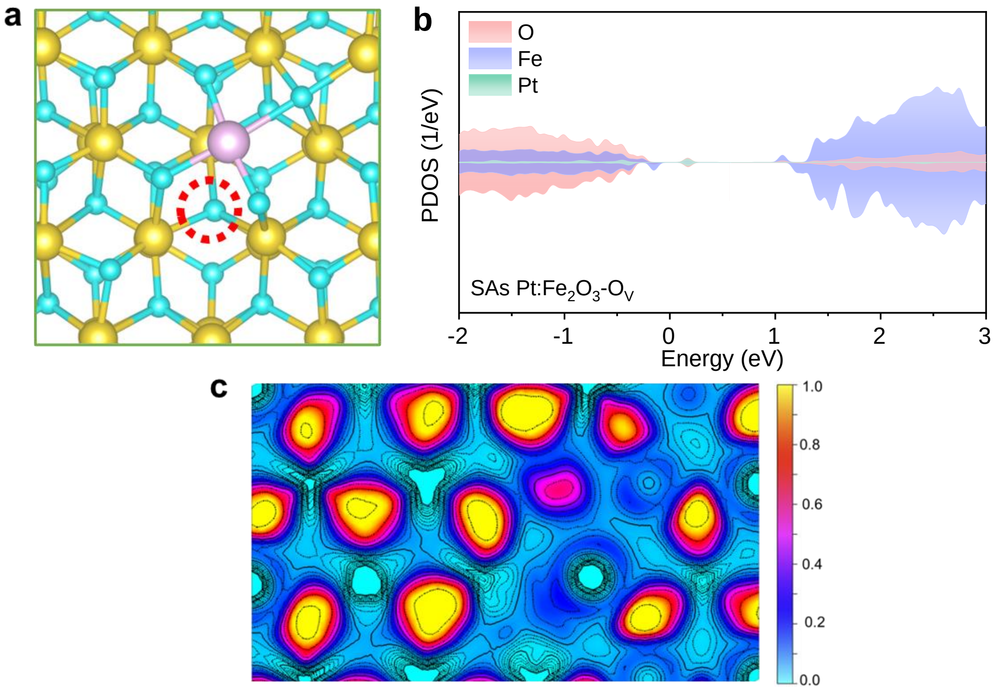

Supplementary Fig. 39 **a** Structural mode, **b** PDOS spectrum, and **c** top view of electron density distribution plot of SAs Pt:Fe_2O_3-O_V.

Discussion: Experiments in this work show that the low power density and short treatment time only induce surface oxygen vacancies. Based on experiments, the SAs Pt:Fe_2O_3-O_V system (Supplementary Fig. 39 a) was built by removing an oxygen atom on the surface of SAs Pt:Fe_2O_3 system. To eliminate interaction between slabs, we added 15 Å vacuum spacing normal to the plane direction.

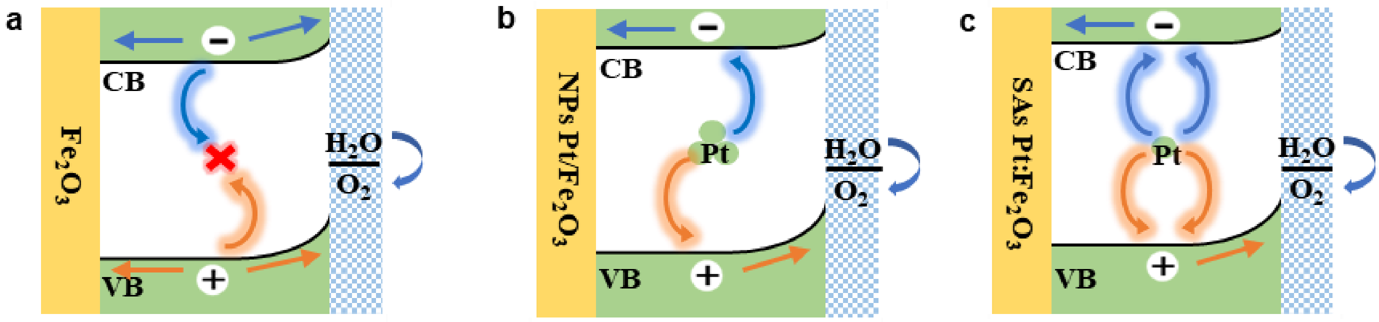

Supplementary Fig. 40 a-c Band bending schematics for **a** Fe_2O_3, **b** NPs Pt/Fe_2O_3, and **c** SAs Pt:Fe_2O_3.

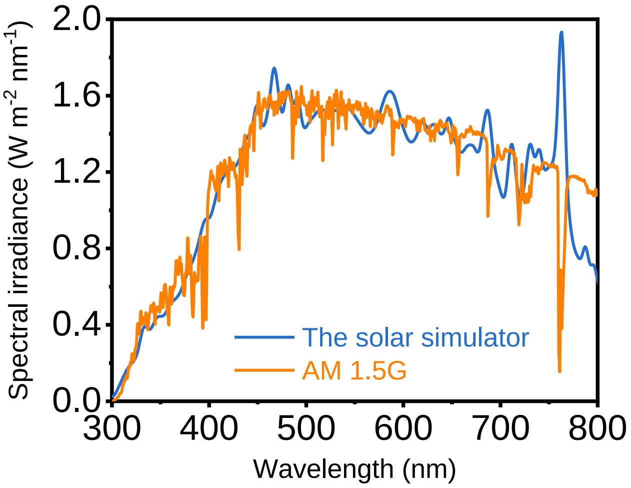

Supplementary Fig. 41 The spectrum comparison of solar simulator with AM 1.5G simulated solar light (ASTM G173-03).

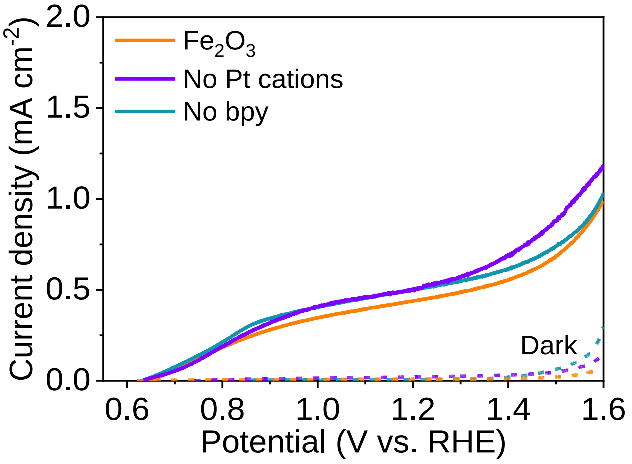

Supplementary Fig. 42 *J-V* curves of Fe_2O_3, PtO_2/Fe_2O_3 and bpy/Fe_2O_3 in 1 M KOH under AM 1.5G illumination (100 mW cm^{-2}).

Discussion: The Fe_2O_3 by using 2,2-bipyridine without the Pt cations (No Pt cations) and the Fe_2O_3 with the Pt cations without using 2,2-bipyridine (No bpy) were also prepared in the smiliar manner, and the related photocurrent densities do not have obvious change comparable to the pristine Fe_2O_3, indicating the important role of single atom Pt in bulk material for the enhanced PEC performance.

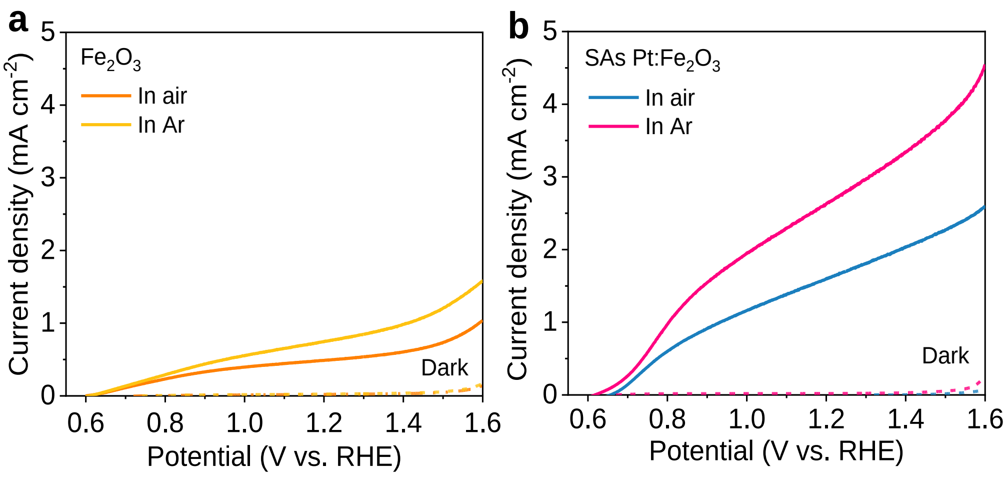

Supplementary Fig. 43 a, b *J-V* curves of **a** Fe_2O_3 and **b** SAs Pt:Fe_2O_3 in second-annealing treatment in air and Ar conditions. All electrochemical measurements were performed in 1 M KOH under AM 1.5G illumination (100 mW cm^{-2}).

Discussion: To better analysis single atomic Pt on Fe_2O_3, Ar annealing treatment is necessary for the separation of Pt atom, avoiding the aggregation of Pt nanoparticles. From Supplementary Fig. 43, we can find that the PEC performance of the SAs Pt:Fe_2O_3 annealing in air is definitely lower than that of Ar condition. This photoresponse in air is closed to the result of NPs Pt/Fe_2O_3 (Fig. 4a). That means that air annealing only induces to the traditional Pt dopant into Fe_2O_3.

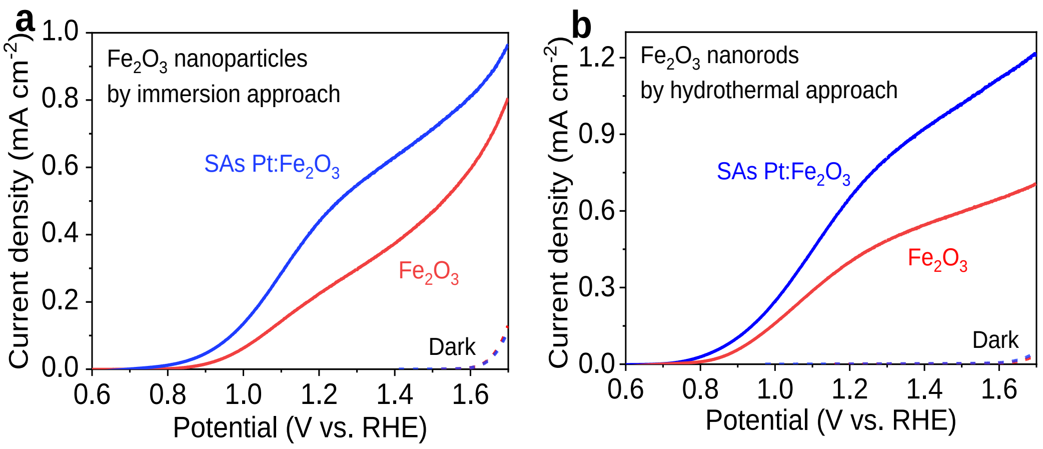

Supplementary Fig. 44 a, b *J-V* curves of SAs Pt:Fe_2O_3 nanoparticles **a** and nanorods **b**.

Discussion: The various nanostructures (nanoparticles and nanorods) of Fe_2O_3 were formed by immersion approach and hydrothermal approaches. The FTO substrate was soaked in 25 mmol of $Fe(NO_3)_3$ at 90 °C for 3 h, followed by thermal treatment at 600 °C for 30 min and 800 °C for 20 min to form the Fe_2O_3 nanoparticles. Fe_2O_3 nanorods were prepared by a hydrothermal approach. FeOOH nanorods on FTO substrates were firstly grown in a solution containing 150 mM ferric chloride hexahydrate, 1 M sodium nitrate, and 40 µL of concentrated nitric acid (70%). Then, FeOOH nanorods were thermally annealed at 600 °C for 30 min and 800 °C for 20 min to form Fe_2O_3. Further Pt single atoms were doped into these structures by using the same procedure with SAs Pt:Fe_2O_3 nanoflakes. No doubt that enhanced PEC activities are attained for single-atom doping. It can be stated that the benefit of one-dimensional ultrathin nanoflakes nanostructure used in this work, which can facilitate charge transfer to the surface and to the back side for oxygen and hydrogen reaction on PEC water splitting.

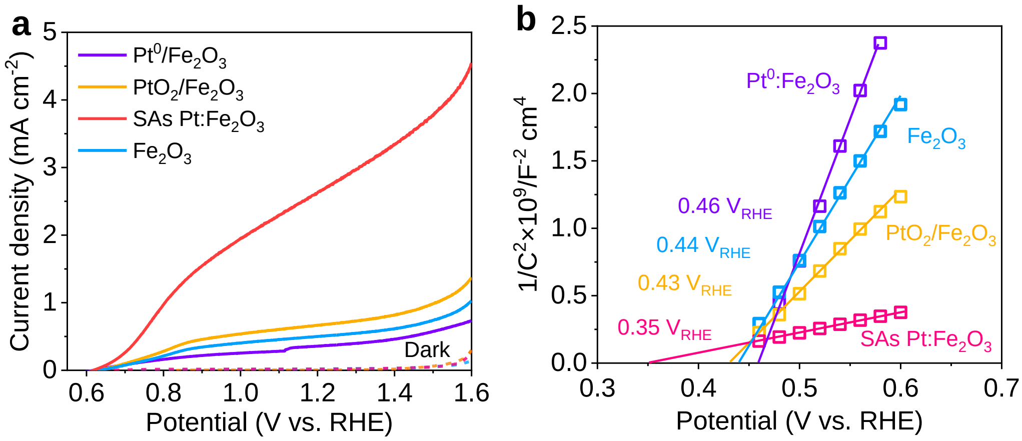

Supplementary Fig. 45 a *J-V* curves and **b** Mott-Schottky plots for Fe_2O_3, Pt^0/Fe_2O_3, PtO_2/Fe_2O_3, and SAs $Pt:Fe_2O_3$.

Discussion: To illustrate the role of single atoms Pt, Pt^0/Fe_2O_3, and PtO_2/Fe_2O_3 were prepared for comparison. Fe_2O_3 was immersed in 5 mmol ethanol solution of hexachloroplatinic acid hexahydrate, followed by 5 min of light irradiation for the synthesis of Pt^0/Fe_2O_3. For the preparation of PtO_2/Fe_2O_3, Pt^0/Fe_2O_3 was annealed at 350 °C in air. The Fe_2O_3 loaded with Pt^0 and Pt^{4+} exhibit the low PEC performances in contrast to SAs $Pt:Fe_2O_3$. This also highlights the advantage of Pt doping in forms of single atoms.

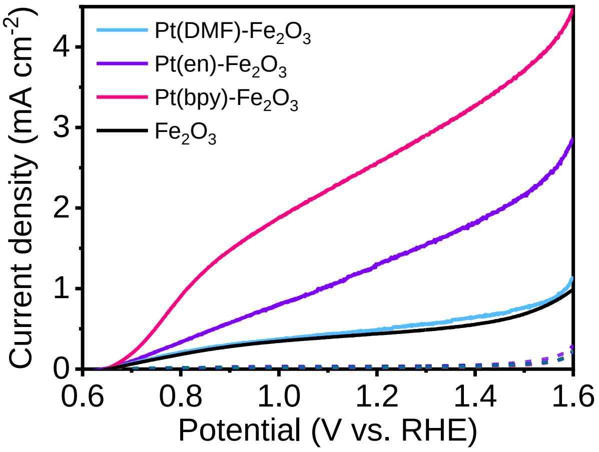

Supplementary Fig. 46 *J-V* curves for Pt species induced Fe_2O_3 with various ligands to chelate the Pt cations: N,N-dimethylformamide (DMF), ethanediamine (en), 2,2-bipyridine (bpy). The measurements were performed in 1 M KOH under AM 1.5G illumination (100 mW cm^{-2}).

Discussion: We compare en and bpy ligands to chelate the Pt species, and both Pt(en) and Pt(bpy) on Fe_2O_3 display the improved PEC performances compared to the pristine Fe_2O_3. No obvious change for Pt(DMF)-Fe_2O_3 is observed with the pristine Fe_2O_3, which means that DMF does not influence much on PEC performance.

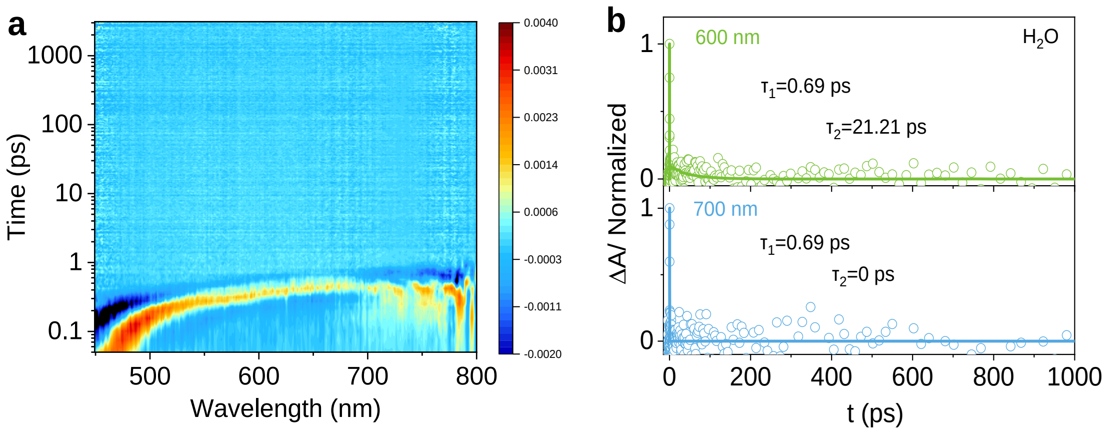

c

Sample	A_1	τ_1 (ps)	A_2	τ_2 (ps)	τ_{av} (ps)
H_2O 600 nm	0.002	0.69	0.0001	21.21	1.67
H_2O 700 nm	0.001	0.69	0	0	0.69

Supplementary Fig. 47 a Time-resolved transient absorption spectra of H_2O when excited with 380 nm light; **b** transient absorption decays observed at 600 nm and 700 nm shown as the solid lines, and the circles represent experimental data; **c** fits for the decays calculated with two exponential decay model of H_2O.

Discussion: To remove the disturbance from the water, the TAS spectrum of water was measured, and it shows a $\tau 1$ value at the short time of less than 1 ps assigned to the effect of water.

Supplementary Table 1 Summary of recent progress of doped Fe_2O_3 photoanodes.

Photoanode	Onset potential (V_{RHE})	J (mA cm^{-2}) @1.23 V_{RHE}	ABPE (%)	Reference
**SAs Pt:Fe_2O_3-O_V**	**0.627**	**3.65**	**0.68**	**This work.**
Ge:Fe_2O_3	0.70	3.50	---	Nat. Commun. 12, 4309 (2021).
Ta:Fe_2O_3@ Fe_2O_3	0.63	2.45	0.31	Nat. Commun. 11, 4622 (2020).
Si:Fe_2O_3	0.9	2.20	---	J. Am. Chem. Soc. 128, 15714 (2006).
Ti:Fe_2O_3	0.95	1.25	---	Angew. Chem. Int. Ed. 55, 9922 (2016).
Zr:Fe_2O_3	0.85	1.38	0.11	Angew. Chem. Int. Ed. 56, 4150 (2017).
P:Fe_2O_3	0.80	2.70	---	Energy Environ. Sci. 8, 1231 (2015).
Ti:Fe_2O_3	0.95	0.92	---	Adv. Funct. Mater. 26, 4414 (2016).
Sn:Fe_2O_3	0.95	0.40	0.02	Adv. Funct. Mater. 28, 1804472 (2018).
Sn:Fe_2O_3	0.80	1.38	---	Nano Lett. 17, 2490 (2017).
Sn:Fe_2O_3	0.65	1.24	---	Nano Lett. 11, 2119 (2011).
Ge:Fe_2O_3	0.85	2.19	---	Nano Energy 9, 282 (2014).
P:Fe_2O_3	0.80	1.48	0.12	Chem. Sci. 8, 91 (2017).
Nb,Sn:Fe_2O_3	0.80	1.88	---	Chem. Sci. 10, 10436 (2019).
Ti:Fe_2O_3	0.71	1.10	---	Nano energy 76, 105089 (2020).

Supplementary Table 2 Structural parameters extracted from Pt-$L3$ edge $\chi(R)$ space spectra fitting of SAs Pt:Fe_2O_3.

	Reduced Chi-square (χ_v^2)	R-factor (%)	amp/S_0^2	$N_{(Pt\text{-}O\ path)}$	$R_{(Pt\text{-}O\ path)}$ (Å)	$\sigma^2_{(Pt\text{-}O\ path)}$ (10^{-3}Å^2)	ΔE_0 (eV)
SAs Pt:Fe_2O_3	927.53	0.0504	0.88+/- 0.09	4.5	1.936± 0.044	2.9+/-1.3	1.66+/-0.98
			amp/ S_0^2	$N_{(Pt\text{-}O\text{-}Fe\ path)}$	$R_{(Pt\text{-}O\text{-}Fe\ path)}$ (Å)	$\sigma^2_{(Pt\text{-}O\text{-}Fe\ path)}$ (10^{-3}Å^2)	ΔE_0 (eV)
			1.06+/- 0.11	1	2.839± 0.101	4.4+/-1.9	2.61+/-1.17

Supplementary Table 3 Fitting data of the PEIS on Fe_2O_3, NPs Pt/Fe_2O_3, and SAs Pt:Fe_2O_3 derived from **Fig. 4f**.

Sample	Rs (Ω)	Rct (Ω)
Fe_2O_3	25.72	2719.40
NPs Pt/Fe_2O_3	22.53	809.80
SAs Pt:Fe_2O_3	23.07	546.50

Supplementary Table 4 Bandgaps and band positions of pristine Fe_2O_3, NPs Pt/Fe_2O_3, and SAs Pt:Fe_2O_3 determined by UPS spectra and UV-vis absorption spectra.

Sample	E_{BG} (eV)	E_F (eV)	E_{VB} (eV)	E_{CB} (eV)
Fe_2O_3	2.07	-4.98	-6.69	-4.62
NPs Pt/Fe_2O_3	2.03	-5.15	-6.76	-4.73
SAs Pt:Fe_2O_3	2.01	-5.23	-6.78	-4.77

Supplementary Table 5 The fits for the decays calculated with three exponential decay model of Fe_2O_3, NPs Pt/Fe_2O_3, and SAs $Pt:Fe_2O_3$.

Sample	A_1×1000	τ_1 (ps)	A_2×1000	τ_2 (ps)	A_3×1000	τ_3 (ps)	τ_{av} (ps)
Fe_2O_3 600 nm	0.75	0.82	0.46	103.67	0.38	1146.92	304.49
Fe_2O_3 700 nm	6.64	0.95	1.47	21.97	1.72	196.63	38.33
NPs Pt/Fe_2O_3 600 nm	1.53	0.59	0.92	185.46	0.95	1384.45	437.28
NPs Pt/Fe_2O_3 700 nm	1.39	0.64	0.44	57.62	0.24	396.45	58.64
SAs $Pt:Fe_2O_3$ 600 nm	1.65	0.80	0.14	252.72	0.82	1504.58	486.76
SAs $Pt:Fe_2O_3$ 700 nm	0.87	0.65	0.12	143.63	0.52	418.36	155.86

Supplementary Table 6 Canonically averaged energy gap, absolute value of NA coupling, pure-dephasing time, and nonradiative charge trapping, detrapping, transfer and recombination times in the Fe_2O_3, NPs Pt/Fe_2O_3, and SAs $Pt:Fe_2O_3$ systems.

Sample		gap (eV)	NA coupling (meV)	dephasing (fs)	time (ps)
Fe_2O_3	CBM-VBM	1.72	1.61	8.60	757
NPs Pt/Fe_2O_3	CBM-VBM	1.02	5.70	13.10	77
	CBM-CBM-2	0.19	42.49	7.90	0.10
SAs $Pt:Fe_2O_3$	CBM-VBM	1.51	2.47	8.50	136
	CBM-e_{trap}	0.08	29.44	23.80	0.2[a]/0.4[b]
	VBM-h_{trap}	0.16	9.98	10.00	0.8[a]/1.1[b]

[a]The charge trapping time. [b]The charge detrapping time.

Supplementary Table 7 Structural parameters extracted from Pt-*L*3 edge $\chi(R)$ space spectra fitting of SAs Pt:Fe_2O_3-Ov.

	Reduced Chi-square (χ_v^2)	*R*-factor (%)	amp/S_0^2	$N_{(Pt\text{-}O\ path)}$	$R_{(Pt\text{-}O\ path)}$ (Å)	$\sigma^2_{(Pt\text{-}O\ path)}$ (10^{-3}Å^2)	ΔE_0 (eV)
SAs Pt:Fe_2O_3-O_V	850.07	0.0288	0.92+/- 0.11	4	1.955± 0.104	2.9+/-1.4	1.97+/-1.32
			amp/ S_0^2	$N_{(Pt\text{-}O\text{-}Fe\ path)}$	R $_{(Pt\text{-}O\text{-}Fe\ path)}$ (Å)	$\sigma^2_{(Pt\text{-}O\text{-}Fe\ path)}$ (10^{-3}Å^2)	ΔE_0 (eV)
			1.05+/- 0.13	1	2.862± 0.112	4.1+/-2.1	3.36+/-1.84

Supplementary Table 8 Atomic element ratios of SAs Pt:Fe_2O_3 and SAs Pt:Fe_2O_3-O_V.

Element	Fe (at.%)	O (at.%)	Pt (at.%)
SAs Pt:Fe_2O_3	22.86	67.17	9.96
SAs Pt:Fe_2O_3-O_V	28.31	64.75	6.97

Supplementary Table 9 Summary of recent progress of Fe_2O_3 photoanodes with cocatalyst decoration.

Photoanode	Onset pontential (V_{RHE})	J (mA cm^{-2}) @1.23 V_{RHE}	ABPE (%)	Reference
**SAs Pt:Fe_2O_3-Ov**	**0.627**	**3.65**	**0.68**	**This work.**
$NiFe(OH)_x$/Ta:Fe_2O_3@Fe_2O_3	0.55	3.22	0.55	Nat. Commun. 11, 4622 (2020).
Co-Pi/Ti:Fe_2O_3	0.86	3.50	---	Nat. Commun. 10, 4832 (2019).
$NiFeO_x$/Dual-regrowth Fe_2O_3	0.45	1.30	---	Nat. Commun. 6, 7447 (2015).
Co-Pi/Si:Fe_2O_3	0.82	2.70	---	J. Am. Chem. Soc. 128, 15714 (2006).
FeOOH/Fe_2O_3	0.65	1.21	---	Angew. Chem. Int. Ed. 55, 10854 (2016).
Ti-SiO_x/CoPi/Ti:Fe_2O_3	0.80	3.19	---	Angew. Chem. Int. Ed. 55, 9922 (2016).
Co-Pi/Zr:Fe_2O_3	0.65	2.20	0.23	Angew. Chem. Int. Ed. 56, 4150 (2017).
Co-Pi/P:Fe_2O_3	0.75	3.10	---	Energy Environ. Sci. 8, 1231 (2015).
FeNiOOH/Fe_2TiO_5/Fe_2O_3	0.95	2.20	---	Energy Environ. Sci. 10, 2124 (2017).
Co-Pi/NPs Ag/Fe_2O_3	0.70	4.68	0.55	Adv. Mater. 28, 6405 (2016).
FeOOH/SnO_2-SiO_x/Ti:Fe_2O_3	0.64	1.54	---	Adv. Energy Mater. 6, 1501840 (2016).
FeOOH/Fe_2O_3	0.80	0.85	---	Adv. Funct. Mater. 25, 2686 (2015).
$CoFeO_x$/Ti:Fe_2O_3	0.78	2.49	---	Adv. Funct. Mater. 26, 4414 (2016).
Fe_2TiO_5/Fe_2O_3/Pt	0.80	1.00	---	Adv. Funct. Mater. 27, 1703527 (2017).
Co-Mn/Fe_2O_3	0.60	2.09	0.25	Adv. Funct. Mater. 29, 1904622 (2019).
FeOOH/$FeTaO_4$/Fe_2O_3	0.80	2.86	0.34	Adv. Funct. Mater. 29, 1805737 (2019).
Co-Pi/Co_3O_4/Ti:Fe_2O_3	0.64	2.70	0.43	Adv. Funct. Mater. 29, 1801902 (2019).
Co-Pi/Fe_2O_3@Fe_2TiO_5	0.90	2.60	---	ACS Nano 9, 5348 (2015).

Cobail oxide/Sn:Fe_2O_3	0.80	2.20	---	Nano Lett. 17, 2490 (2017).
M:B-Fe_2O_3	0.83	2.35	---	ACS Catal. 8, 11932 (2018).
FeOOH/Ti,Sn:Fe_2O_3/B:Fe_2O_3	0.84	2.35	---	ACS Catal. 8, 11932 (2018).
$NiFeO_x$/Nb,Sn:Fe_2O_3@$FeNbO_4$	0.71	2.71	0.45	ACS Catal. 9, 1289 (2019).
Ru-Fe_2O_3	0.71	5.7	---	Nano Energy 16, 320 (2015).
Au/FeOOH/Fe_2O_3	0.60	3.20	0.47	Nano Energy 35, 171 (2017).
Co/Pi/H_2 treatment TiO_2/Fe_2O_3	0.55	6.00	---	Nano Energy 39, 211 (2017).
FeOOH/Sn-D-Fe_2O_3 NFs	0.60	2.40	0.27	Nano Energy 50, 331 (2018).
$NiFeO_x$@Ti:Si-Fe_2O_3	0.71	2.62	---	Nano Energy 76, 105089 (2020).
Co-Pi/Fe_2O_3	0.70	2.00	0.32	Chem. Sci. 8, 91 (2017).
$NiFeO_x$/Fe_2O_3@Fe_2TiO_5	0.80	2.70	---	Small 12, 3415 (2016).
Sb:SnO_2/Fe_2O_3	0.70	1.50	---	Small 14, 1703860 (2018).

References:

1. Fampiou, I., Ramasubramaniam. CO adsorption on defective graphene-supported Pt_{13} nanoclusters. *J. Phys. Chem. C* **117**, 19927-19933 (2013).
2. Boronat, M., Corma, A. Origin of the different activity and selectivity toward hydrogenation of single metal Au and Pt on TiO_2 and bimetallic Au-Pt/TiO_2 Catalysts. *Langmuir* **26**, 16607-16614 (2010).
3. Xiao, F., Wang, Y., Xu, G.-L., Yang, F., Zhu, S., Sun. C.-J., Cui, Y., Xu. Z., Zhao, Q., Jang, J., Qiu, X., Liu, E., Drisdell, W. S., Wei, Z., Gu, M., Amine, K., Shao, M. Fe-N-C boosts the stability of supported platinum nanoparticles for fuel cells. *J. Am. Chem. Soc.* **144**, 20372-20384 (2022).
4. Zaban, A., Greenshtein, M. & Bisquert, J. Determination of the electron lifetime in nanocrystalline dye solar cells by open-circuit voltage decay measurements. *ChemPhysChem* **4**, 859-864 (2003).
5. Zhang, H., Li, D., Byun, W. J., Wang, X., Shin, T. J., Jeong, H. Y., Han, H., Li, C., Lee, J. S. Gradient tantalum-doped hematite homojunction photoanode improves both photocurrents and turn-on voltage for solar water splitting. *Nat. Commun.* **11**, 4622 (2020).
6. Takata, T., Hitoki, G., Kondo, J. N., Kobayashi, H., Domen, K. Visible-light-driven photocatalytic behavior of tantalum-oxynitride and nitride. *Res. Chem. Intermed.* **33**, 13-25 (2007).
7. Hou, Y. Du, X., Scheiner, S., Mimeekin, D. P., Wang, Z., Li, N., Killian, M. S., Chen, H., Richter, M., Levchuk, I., Schrenker, N., Spiecker, E., Stubhan, T., Luechunger, N. A., Hirsch, A., Schmuki, P., Steinruch, H.-P., Fink, R. H., Halik, M., Snaith, H. J., Brabec, C. J. A generic interface to reduce the efficiency-stability-cost gap of perovskite solar cells. *Science* **358**, 1192-1197 (2017).
8. Xiao, Y., Feng, C., Fu, J. Wang, F., Li, C., Kunzelmann, V F., Jiang, C.-M., Nakabayashi, M., Shibata, N., Sharp, I. D., Domen, K. Band structure engineering and defect control of Ta_3N_5 for efficient photoelectrochemical water oxidation. *Nat. Catal.* **3**, 932-940 (2020).
